# The prognostic value of NLRP1/NLRP3 and its relationship with immune infiltration in human gastric cancer

**DOI:** 10.18632/aging.204438

**Published:** 2022-12-19

**Authors:** Ping Wang, Yulan Gu, Jianke Yang, Jiamin Qiu, Yeqiong Xu, Zengxiang Xu, Jiguang Gao, Chuandan Wan

**Affiliations:** 1 School of Preclinical Medicine, Wannan Medical College, Wuhu 241001, China; 2 Department of Oncology, Changshu Second People’s Hospital, Changshu 215500, China; 3 Department of Pathology, Changshu Second People’s Hospital, Changshu 215500, China; 4 Central laboratory of Changshu Medical examination Institute, Changshu 215500, China

**Keywords:** inflammasome, NLR family pyrin domain containing protein, gastric cancer, immune cell infiltration

## Abstract

Background: Inflammasomes are related to tumorigenesis and immune-regulation. Here, we investigated the prognostic value of the NLR family pyrin domain containing (NLRP) 1/NLRP3 inflammasome and its potential mechanisms in immune-regulation in gastric cancer (GC).

Methods: We analyzed the differential expression of NLRP1/NLRP3 between tumor and normal tissues using the Oncomine and Tumor Immune Estimate Resource (TIMER) databases. Immunohistochemistry and western blotting were used to detect NLRP1/NLRP3 protein expression in GC tissues. Correlations between NLRP1/NLRP3 expression levels and patient survival were analyzed using Kaplan-Meier survival curves. The relationships of NLRP1/NLRP3 expression and tumor-infiltrating immune cells/marker genes were assessed using the TIMER database. NLRP1/NLRP3 and immune checkpoint gene correlations were verified by single-gene co-expression analyses, and tumor immune-related pathways involving NLRP1/NLRP3 were analyzed using gene set enrichment analysis (GSEA).

Results: Elevated NLRP1/NLRP3 expression was significantly correlated with lymph node metastasis, poor survival, immune-infiltrating cell abundances, and immune cell markers. NLRP3 showed stronger correlations with immune infiltration and the prognosis of gastric cancer. NLRP1 and NLRP3 might be involved in the same tumor immune-related pathways. Thus, high NLRP1/NLRP3 expression promotes immune cell infiltration and poor prognosis in GC. NLRP1/NLRP3, particularly NLRP3, may have important roles in immune infiltration and may serve as a prognostic biomarker for GC.

Conclusions: NLRP1/NLRP3, particularly NLRP3, may have important roles in immune infiltration and may serve as a prognostic biomarker for GC.

## INTRODUCTION

Gastric cancer (GC) is the third-leading cause of cancer-related death worldwide [1]. According to the latest Global Burden of Cancer data for 2020, China had 478,000 new cases of GC in 2020, accounting for 43.9% of new cases worldwide, and 373,000 GC-related deaths, accounting for 48.5% of global deaths [2]. Despite improvements in detection techniques and cancer-related treatments, most patients already have late-stage GC at the time of diagnosis, and the lack of screening techniques hampers early diagnosis, leading to a poor 5-year survival rate [3]. To improve the screening and diagnostic rate for GC, there is a need for accurate and sensitive biomarkers.

Immunotherapy has made great strides in the past decade. Unlike targeting the tumor itself, immunotherapy aims to overcome immune suppression caused by the tumor microenvironment, enabling the innate immune system to target and kill cancer cells [4]. Immunotherapy based on immune checkpoint inhibitors has led tumor therapy into a new era [5, 6]. However, identifying predictive biomarkers is necessary for achieving precise tumor immunotherapy [7].

Nucleotide-binding domain and leucine-rich repeat pyrin-domain-containing protein (NLRP) forms the inflammasome, which can play crucial roles in innate immunity and inflammation [8]. As an inflammatory-response sensor signaling protein, NLRP mediates the activation of caspases to induce cytokine maturation and pyroptosis [9]. Different NLRP members are expressed in different organ tissues and are involved in various cancers, regulating innate and adaptive immune responses, cell death, and cell proliferation via different pathways [10]. NLRP1 and NLRP3 are the most important components of inflammasomes and involved in distinct regulatory mechanisms [9]. Some cytokines, such as IL-1β precursor, depends on the activation of cytoplasmic caspase hydrolases, which are activated by NLRP inflammasomes, particularly NLRP1 and NLRP3 [11]. NLRP3 activates caspases through the adaptor protein ASC and promotes IL-1β and IL-18 secretion, whereas NLRP1 possesses a caspase recruitment domain, can directly bind to caspase proteins, and does not require ASC for transconjugation [8].

The multiple effects of NLRP1 and NLRP3 are closely related to various human diseases associated with dysfunctional immunoregulation, including autoimmune diseases [12], asthma [13], psoriasis [14], lupus nephritis [15], and Alzheimer’s disease [16], and have also been reported to play crucial roles in tumor progression, prognosis, and treatment response [17–19]. However, due to the tissue-dependent or cell-dependent roles of inflammasomes in tumorigenesis, the expression levels of inflammasome-related genes vary, and these genes play various roles in different cancers [20]. Inflammasome components can prevent tumor development or promote tumor development in some types of cancers [21, 22]. For example, activation of NLRP3 can promote tumor progression and metastasis in breast cancer [23, 24], and the NLRP3 inflammasome can induce cell proliferation, invasion, and tumor development in GC cells [25, 26]. However, NLRP3 functions as a tumor suppressor and protects against colitis-associated cancer [27]. Furthermore, NLRP3 also promotes the anticancer activity of natural killer cells and inhibits liver metastasis in colorectal cancer [28]. Considering the potentially important role of inflammasomes in tumorigenesis and immune regulation and functional differences among inflammasome family members, it is necessary to analyze the relationships between different inflammasomes and specific tumors. However, there have been no studies on the relationships of the NLRP1/NLRP3 inflammasome with prognosis and immune infiltration in GC.

Therefore, in this study, we analyzed the correlations between NLRP1/NLRP3 and prognosis in patients with GC using experimental data and public databases, such as Oncomine, Tumor Immune Estimate Resource (TIMER), The Cancer Genome Atlas (TCGA), and Kaplan-Meier Plotter. In addition, we also investigated the correlation of NLRP1/NLRP3 expression levels with clinicopathological characteristics and infiltrating immune cells. Gene set enrichment analysis (GSEA) was conducted to explore the immune-related pathways in which NLRP1/NLRP3 may participate during the regulation of GC. Overall, our findings provided preliminary evidence of an association between NLRP1/NLRP3 and immune cell infiltration in GC and afforded insights into the mechanisms through which NLRP1 and NLRP3 regulate this process.

## METHODS

### Analysis of NLRP1/NLRP3 mRNA expression

The differential expression of *NLRP1/NLRP3* mRNA in various tumors and normal tissues was analyzed using the Oncomine and TIMER databases. Oncomine is a platform for cancer gene information mining, based on a cancer gene chip database. TIMER is a comprehensive platform for tumor-infiltrating immune cell analysis in 32 cancer types (https://cistrome.shinyapps.io/timer/). The Diff Exp module in TIMER was used to analyze differential gene expression. In Oncomine, we selected data showing 1.5-fold changes in expression and a *p* value of less than 0.05 for differences in mRNA levels. NLRP1/NLRP3 differential expression in primary gastric cancer and tumor was evaluated by using TCGA data in UALCAN (http://ualcan.path.uab.edu/index.html).

### Immunohistochemistry

We collected 20 cases of primary GC and corresponding adjacent normal tissue samples and performed immunohistochemical staining. Briefly, GC sections were incubated with monoclonal antibodies against NLRP1/NLRP3 (all from Abcam Company, Cambridge, UK) at a 1:100 dilution overnight at 4°C. The sections were conjugated with horseradish peroxidase-conjugated antibodies (Abcam) at a 1:500 dilution for 2 h at room temperature. Then 3,3-diaminobenzidine was added. The slides were mounted with Vectashield mounting medium. Light microscopy (Olympus) was used to observe the samples. Immunohistochemical scores were determined independently by two pathologists as previously described [29]. The experimental samples were collected from September to December 2021 in the Oncology Department of Changshu Second People's Hospital (Supplementary Table 1).

### Western blot analysis

Protein was extracted from all tissue samples, and western blotting was carried out according to the methods previously reported in our laboratory [30]. β-Actin was used as a loading control and was detected using specific antibodies (1:1000; Abcam). The other primary antibodies used in this study were rabbit anti-NLRP1 (1:1000; Abcam) and rabbit anti-NLRP3 (1:500; Abcam), and the secondary antibodies were goat anti-rabbit IgG/HRP (1:1000; Sanjian, China). The data were obtained using Quantity One 4.62 software. Three independent experiments were conducted, and all data are represented as means ± standard deviations. Results with *P* values less than 0.05 were considered statistically significant. Statistical analyses and graphs were acquired using GraphPad Prism 8.0 software.

### Prognosis and clinical characteristics analysis

The correlations of NLRP1/NLRP3 expression levels with survival and clinical characteristics in patients with GC were analyzed using Kaplan-Meier Plotter (*n* = 875) (http://www.kmplot.com) and TCGA (*n* = 392) databases. Kaplan-Meier analysis results are presented as survival curves and tables with hazard ratios (HRs) and log-rank *P* values. The results of TCGA analysis were presented as survival curves and bar charts. Results with a *p* value less than 0.05 were regarded as having statistical significance.

### Immune cell infiltration and co-expression analysis

The Gene and Correlation Modules in the TIMER database were used to analyze the correlations of NLRP1/NLRP3 expression with the abundances of six types of tumor-infiltrating immune cell types and the expression levels of 58 immune cell markers [31, 32]. Stomach adenocarcinoma (STAD) RNAseq data from TCGA were used for co-expression correlation analysis between NLRP1/NLRP3 and immune checkpoint genes. Data were analyzed using the graphical visualization software package GGploT2 version 3.3.3 (https://ggplot2.tidyverse.org/). Spearman’s correlation analysis was used to evaluate correlations with gene expression. The strength of the correlation was considered to be significantly positive when the correlation coefficient was greater than 0.3 and the *p* value was less than 0.01.

### GSEA

RNA-seq fpkm data from STAD in TCGA were downloaded from the UCSC Xena site (https://xena.ucsc.edu/). STAD patients were divided into a high-expression group and a low-expression group according to the median NLRP1/NLRP3 expression level. Then, GSEA_4.1.0 software [33] was used for single-gene GSEA analysis. All items were listed in descending order of standardized enrichment score (NES), and the top 10 items related to tumor immunity were listed as the analysis results from the high-expression group; the top 5 items are plotted. Items with a nominal *p* value less than 0.01, false discovery rate *q*-value less than 0.01, and NES greater than 2.0 were regard as significantly enriched.

### Availability of data and material

All datasets generated for this study are included in the article.

## RESULTS

### Expression of NLRP1/NLRP3 in public databases and clinical cases

**Figure 1:**
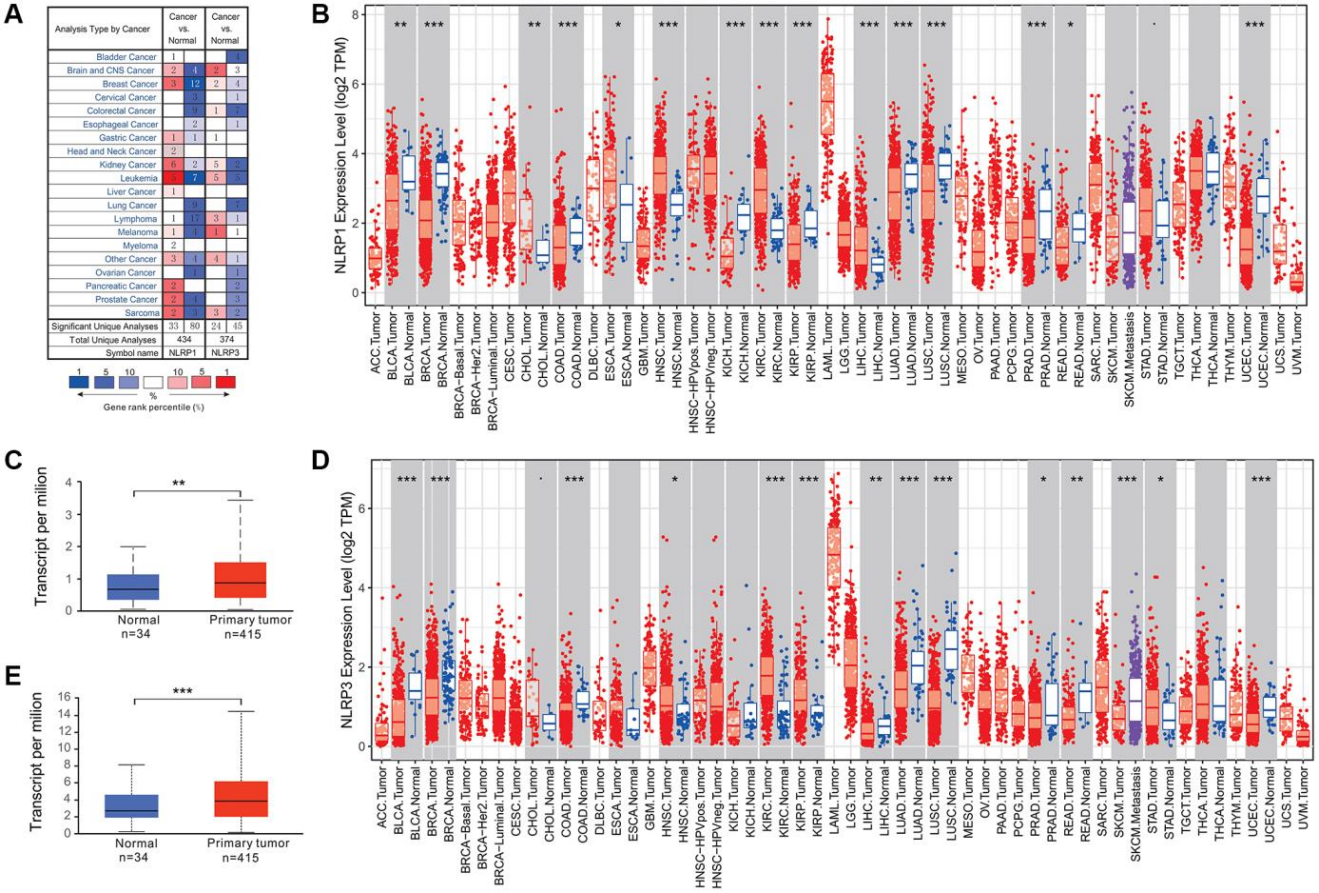
**NLRP1/NLRP3 expression levels in various types of human cancers.** (**A**) NLRP1/NLRP3 expression in data sets of different cancers compared with normal tissues in the Oncomine database. (**B** and **C**) NLRP1/NLRP3 expression levels in various types of cancers were determined by TIMER 2.0. (**D** and **E**) NLRP1/NLRP3 expression levels in primary gastric cancer and normal tissues from TCGA data by UALCAN (^*^p < 0.05, ^**^p < 0.01, ^***^p < 0.001).

Different inflammasome members may play distinct roles in tumorigenesis [20]. To explore the correlations between NLRP1/NLRP3 and tumorigenesis, we analyzed NLRP1/NLRP3 expression differences between various tumors and corresponding normal tissues based on chip data in Oncomine and RNA-seq data in TIMER database. As shown in Figure 1A, NLRP1/NLRP3 expression was varied among datasets for the same tumor type. There were three GC datasets in Oncomine; NLRP1 was upregulated in one dataset and downregulated in one dataset, whereas one dataset showed no difference in NLRP3 expression between tumor and normal tissues (Figure 1A). TIMER analysis showed that NLRP1 and NLRP3 were both downregulated in six tumor tissues types, including bladder urothelial carcinoma (BLCA), breast invasive carcinoma (BRCA), colon adenocarcinoma (COAD), lung adenocarcinoma (LUAD), lung squamous cell carcinoma) (LUSC), and rectum adenocarcinoma (READ), and were both upregulated in tumor tissues of kidney renal clear cell carcinoma (KIRC) and stomach adenocarcinoma (STAD), as compared with corresponding normal tissues (Figure 1B and 1D). UALCAN analysis of TCGA data showed that NLRP1/NLRP3 expression level in primary gastric cancer was significantly higher than normal tissues (Figure 1C and 1E, ^**^p < 0.01, ^***^p < 0.001).

**Figure 2:**
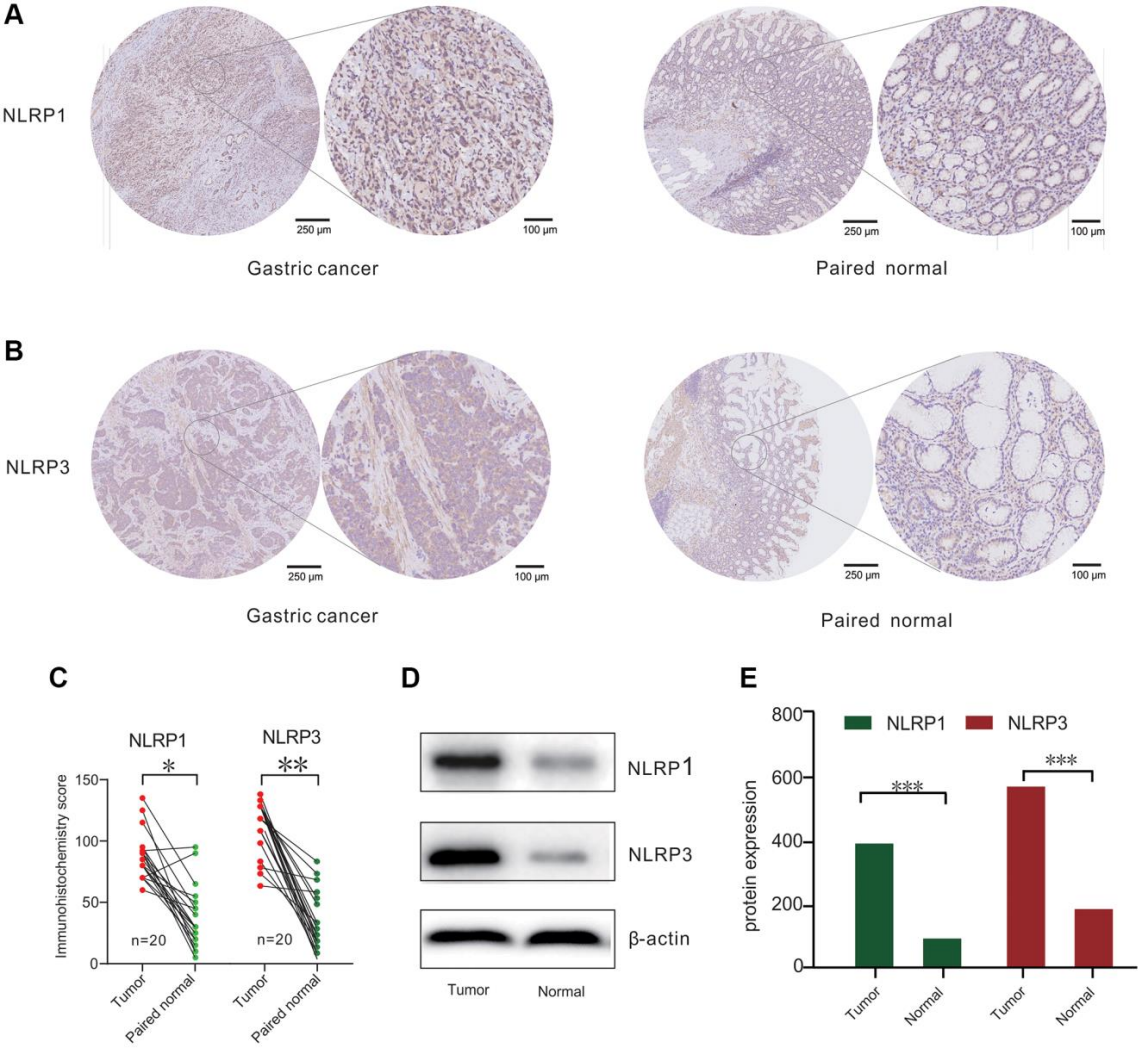
**NLRP1/NLRP3 protein differently expressed in patients with gastric cancer (GC).** (**A** and **B**) Representative Immunohisto-chemistry (IHC) staining for NLRP1 and NLRP3 from 20 gastric tissue and paired normal tissue. (**C**) IHC analysis for NLRP1/NLRP3 protein levels in 20 paired GC cases. (**D**) Representative Western blotting results of NLRP1/NLRP3 in 20 GC cases. (**E**) Histogram of NLRP1/NLRP3 protein expression level from 20 GC cases (^*^p < 0.05, ^**^p < 0.01, ^***^p < 0.001).

To evaluate the expression of NLRP1/NLRP3 protein in GC, we performed immunohistochemical and western blot analysis using 20 paired clinical tissue samples of primary GC. The results showed that NLRP1 and NLRP3 were highly expressed in GC tissues as compared with those in adjacent normal tissues (Figure 2A–2E, *p* < 0.05). Considering both our database analysis and experimental results, we concluded that high expression of NLRP1/NLRP3 may promote the occurrence of GC.

### Potential clinical prognostic value of NLRP1/NLRP3 in GC

**Figure 3:**
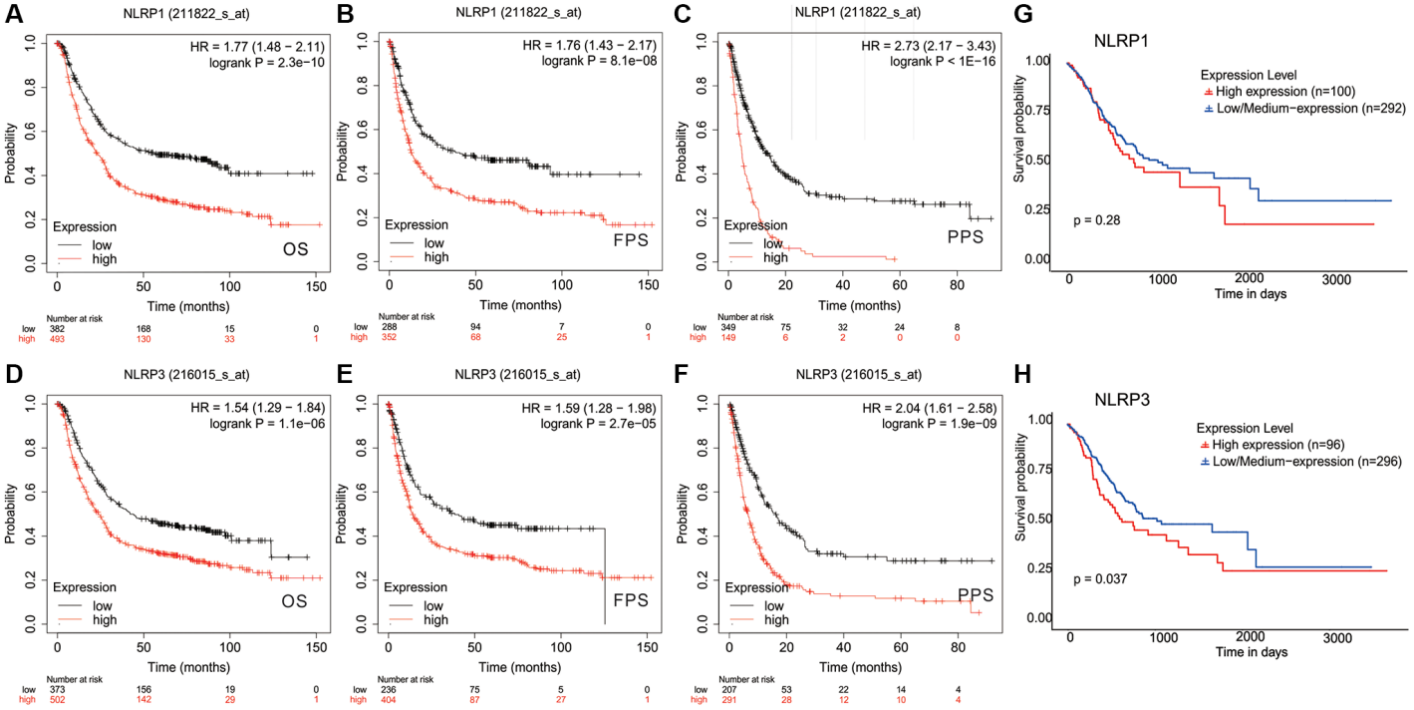
**Kaplan-Meier survival curves comparing the high and low expression of NLRP1/NLRP3 in gastric cancer.** In the Kaplan-Meier plotter database, (**A**–**C**) high NLRP1 expression was correlated with poor OS (*n* = 875, HR = 1.77, *p* = 2.3e-10), FPS (*n* = 640, HR = 1.76, *p* = 8.1e-08), and PPS (*n* = 498, HR = 2.73, *p* = 1e-16); (**D**–**F**) high NLRP3 expression was correlated with bad OS(*n* = 875, HR = 1.54, *p* = 1.1e-06), PFS (*n* = 640, HR = 1.59, *p* = 2.71e-05), and PPS (*n* = 498, HR = 2.04, *p* = 1.9e-09). (**G** and **H**) In TCGA data, High NLRP1/NLRP3 expression was associated with lower 10-year survival (*n* = 392, NLRP1 *p* = 0.28; NLRP3 *p* = 0.037).

Survival analysis was performed to investigate the clinical prognostic potential of NLRP1/NLRP3 in GC. Kaplan-Meier survival analysis of 875 patients with GC revealed that patients with high NLRP1/NLRP3 expression had worse overall survival (OS), first-progression survival (FPS), and post-progression survival (PPS); (Figure 3A–3C; NLRP1: OS, HR = 1.77, *p* = 2.3 × 10^−10^; FPS, HR = 1.76, *p* = 8.1 × 10^−8^; PPS, HR = 2.73, *p* = 1 × 10^−16^; Figure 3D–3F; NLRP3: OS, HR = 1.54, *p* = 1.1 × 10^−6^; FPS, HR = 1.59, *p* = 2.7 × 10^−5^; PPS, HR = 2.04, *p* = 1.9 × 10^−9^), suggesting that high expression of NLRP1/NLRP3 may be related to reduced survival in patients with GC. To validate the above results based on microarray data, we used TCGA RNA-seq data and performed survival analysis in 392 patients with GC. The results showed that patients with high NLRP1/NLRP3 expression had reduced 10-year survival rates (Figure 3G; NLRP1: *p* = 0.28; Figure 3H; NLRP3: *p* = 0.037). From this analysis, we inferred that high NLRP1/NLRP3 expression, particularly high NLRP3 expression, was associated with poor outcomes in patients with GC.

### Correlation of NLRP1/NLRP3 with the clinicopathological characteristics of GC

**Figure 4:**
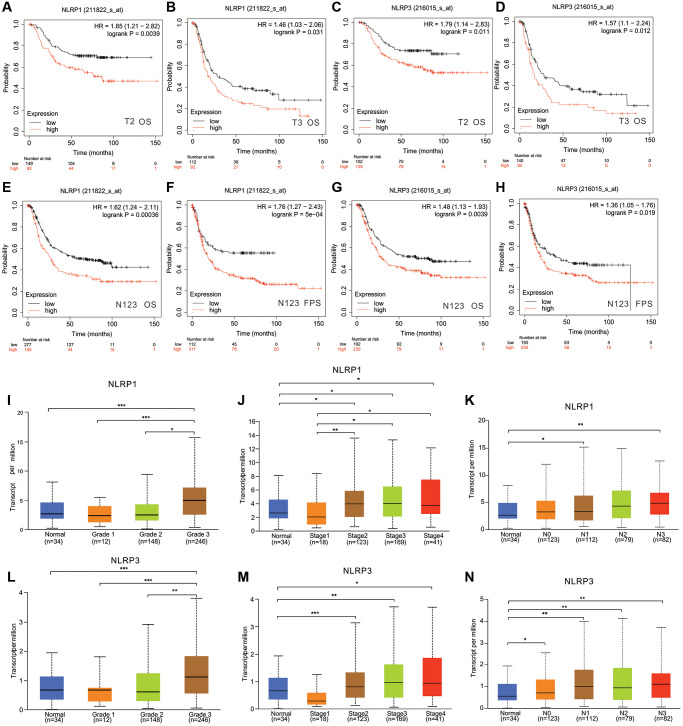
**The relationship between NLRP1/NLRP3 expression levels with clinical characteristics of patients with GC.** In Kaplan Meier Plotter, high expression of NLRP1 was correlated with worse prognosis of GC patients in (**A**) Stage T2 (*n* = 241, OS, HR = 1.85, *p* = 0.0039), (**B**) Stage T3 (*n* = 204, OS HR = 1.461, *p* = 0.031). High expression of NLRP3 was correlated with worse prognosis of GC patients in (**C**) stage T2 (*n* = 241, OS, HR = 1.79, *p* = 0.011), (**D**) Stage T3 (*n* = 204, OS, HR = 1.57, *p* = 0.012). High expression of NLRP1 was correlated with worse prognosis of GC patients in (**E**, **F**) Stages N1+2+3 (*n* = 422, OS, HR = 1.62, *p* = 0.00036; PFS, HR = 1.76, *p* = 5e−04). High expression of NLRP3 was correlated with worse prognosis of GC patients in (**G**, **H**) Stages N1+2+3 (*n* = 422, OS, HR = 1.48, *p* = 0.0039; PFS, HR = 1.36, *p* = 0.019). (**I**–**N**) In TCGA, Expression level of NLRP1/NLRP3 has a significant correlation with tumor grade, cancer stage, and lymph node metastasis. ^*^p < 0.05, ^**^p < 0.01. Notes: Grade 1, Well differentiated (low grade); Grade 2, Moderately differentiated (intermediate grade); Grade 3, Poorly differentiated (high grade); Grade 4, Undifferentiated (high grade). N0, No regional lymph node metastasis; N1, Metastases in 1 to 3 axillary lymph nodes; N2, Metastases in 4 to 9 axillary lymph nodes; N3, Metastases in 10 or more axillary lymph nodes.

We further analyzed the correlation between NLRP1/NLRP3 expression level and different clinicopathological characteristics in 875 patients with GC from Kaplan-Meier Plotter database (Table 1). The expression levels of NLRP1/NLRP3 were significantly correlated with tumor stage and degree in patients with GC (Table 1, Figure 4). Specifically, high expression of NLRP1 was significantly correlated with poor OS in patients with stages 1–3 cancer (Table 1; *p* = 0.048, *p* = 0.004, *p* = 0.0002), whereas high expression of NLRP3 was only correlated with poor outcome in patients with stage 3 GC (OS, *p* = 0.0003; FPS, *p* = 0.021). High expression of NLRP1 and NLRP3 was significantly correlated with reduced OS in patients with stages T2–3 GC (Figure 4A–4D, *p* < 0.05) and stages N1–3 GC (Figure 4E–4H, *p* < 0.01). TCGA data analysis showed similar results. Overall, high NLRP1/NLRP3 expression levels had a more significant correlation with higher tumor grade, cancer stage and more axillary lymph nodes metastases (Figure 4I–4N). These findings suggested that NLRP1 and NLRP3 may play similar roles in the progression of GC, and high NLRP1/NLRP3 expression may be a potential risk factor for the invasion and metastasis of GC *in vivo*, thereby contributing to poor prognosis.

**Table 1:** Association between NLRP1/NLRP3 expression level and clinicopathological characteristics in patients with gastric cancer by Kaplan-Meier plotter.

	**Overall survival**					**First progression survival**				
	***N***	**NLRP1**		**NLRP3**		***N***	**NLRP1**		**NLRP3**	
		**Hazard ratio**	***P*-value**	**Hazard ratio**	***P*-value**		**Hazard ratio**	***P*-value**	**Hazard ratio**	***P*-value**
**SEX**										
Female	236	2.03 (1.43−2.89)	5.6e−05	1.59 (1.20–2.29)	0.012	201	1.95 (1.31−2.9)	0.00075	1.49 (1.02−2.19)	0.037
Male	544	1.88 (1.50−2.35)	1.5e−08	1.92 (1.49–2.49)	3.9e−07	437	1.81 (1.41−2.33)	2e−06	1.74 (1.34−2.27)	3.3e−05
**STAGE**										
1	67	2.64 (0.97−7.18)	0.048	1.97 (0.71–5.47)	0.19	60	0.44 (0.13−1.52)	0.18	0.58 (0.18−1.89)	0.36
2	140	2.35 (1.29−4.28)	0.0041	1.30 (0.69–2.45)	0.41	131	1.71 (0.93−3.12)	0.081	0.64 (0.33−1.25)	0.19
3	305	1.72 (1.29−2.29)	0.00018	1.86 (1.32–2.62)	0.00034	186	2.05 (1.29−3.24)	0.0018	1.56 (1.07−2.29)	0.021
4	148	1.35 (0.88−2.06)	0.16	1.31 (0.9−1.92)	0.16	141	0.83 (0.57−1.22)	0.34	1.31 (0.89−1.92)	0.17
**STAGE T**										
2	241	1.85 (1.21−2.82)	0.0039	1.79 (1.14−2.83)	0.011	239	1.72 (1.14−2.59)	0.0093	1.66 (1.06−2.6)	0.026
3	204	1.46 (1.03−2.06)	0.031	1.57 (1.1−2.24)	0.012	204	1.27 (0.91−1.77)	0.16	1.45 (1.03−2.05)	0.034
4	38	1.32 (0.54−3.23)	0.53	1.32 (0.54−3.23)	0.53	39	1.43 (0.62−3.28)	0.4	2.09 (0.97−4.49)	0.055
**STAGE N**										
0	74	2.05 (0.82−5.1)	0.12	0.49 (0.2−1.19)	0.11	72	2.12 (0.86−5.24)	0.096	0.58 (0.24−1.38)	0.22
1	225	2.23 (1.48−3.36)	8.5e−05	1.31 (0.86−1.98)	0.21	222	1.87 (1.27−2.77)	0.0014	1.23 (0.82−1.83)	0.31
2	121	2.05 (1.27−3.32)	0.0028	2.39 (1.35−4.25)	0.0021	125	1.83 (1.15−2.92)	0.01	2.22 (1.28−3.85)	0.0034
3	76	1.54 (0.88−2.72)	0.13	1.89 (1.07−3.32)	0.026	76	1.35 (0.77−2.36)	0.29	1.81 (1.02−3.2)	0.039
123	422	1.62 (1.24−2.11)	0.00036	1.48 (1.13−1.93)	0.0039	423	1.76 (1.27−2.43)	5e−04	1.36 (1.05−1.76)	0.019
**STAGE M**										
0	444	1.64 (1.24−2.18)	0.00046	1.54 (1.1−2.16)	0.011	443	1.69 (1.23−2.33)	0.001	1.47 (1.06−2.04)	0.02
1	56	1.52 (0.79−2.92)	0.21	1.89 (1.01−3.54)	0.042	56	0.74 (0.39−1.41)	0.35	0.61 (0.33−1.15)	0.12
**DIFFERRATION**										
POOR	165	1.37 (0.89−2.11)	0.15	1.59 (1.05−2.39)	0.025	121	1.5 (0.94−2.4)	0.088	0.82 (0.52−1.29)	0.39
MODERATE	67	1.86 (0.81−4.24)	0.14	0.56 (0.28−1.1)	0.089	67	1.71 (0.79−3.72)	0.17	0.64 (0.33−1.22)	0.17
WELL	32	7.47 (1.72−32.41)	0.0017	2.45 (0.82−7.32)	0.098	5	NA		NA	

Note: T represents the size and invasion of the tumor; T1 indicates that the tumor is confined to the mucosa and submucosa; T2 indicates that the tumor is infiltrated into the muscle layer; T3 indicates that the tumor is infiltrated into the subserous membrane; T4 represents the tumor infiltrating through the serosal layer. N0 indicates no local lymph node metastasis; N1-N3 indicates local lymph node metastasis. M0 means no distant organ metastasis; M1 means distant organ metastasis of gastric cancer.

### Association of the immune cell infiltration with NLRP1/NLRP3 expression and prognosis of GC

**Figure 5:**
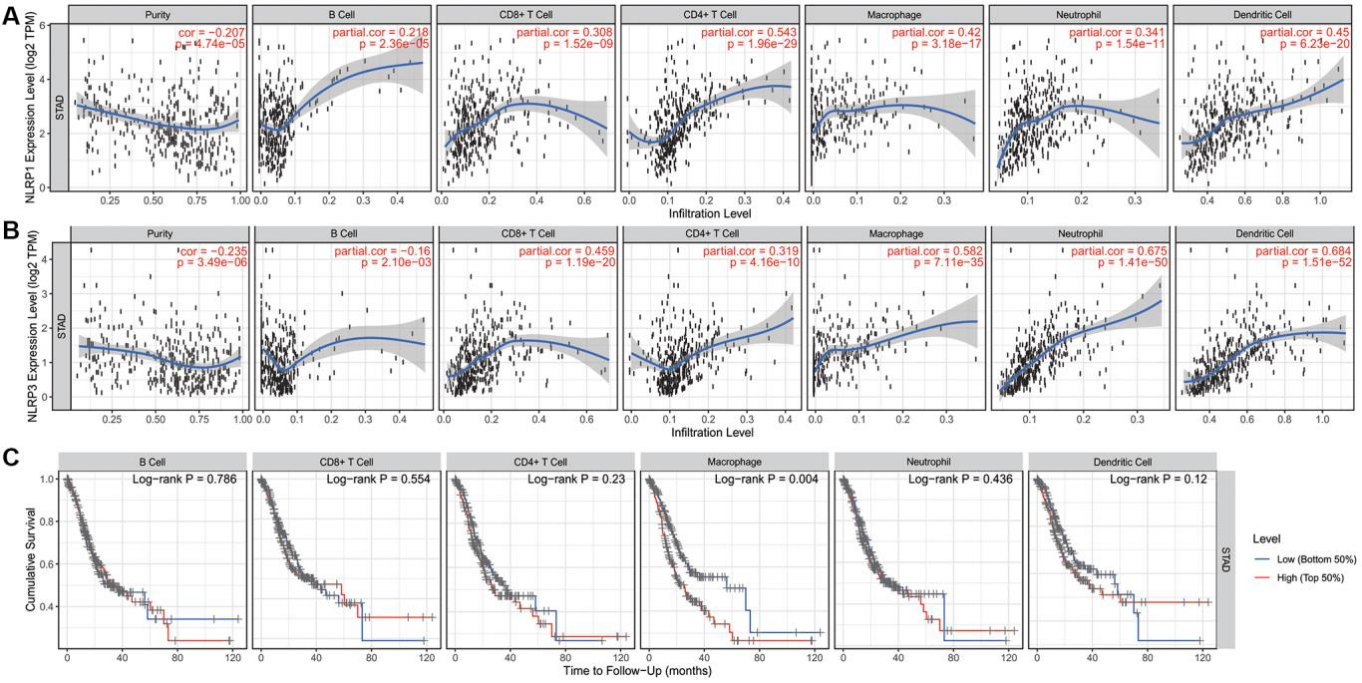
**The correlation of infiltration levels of six types of immune cell types with NLRP1/NLRP3 expression levels and survival in STAD (*n* = 415) in TIMER 2.0.** (**A**) NLRP1 expression level is significantly negatively related to tumor purity and has a significantly positive correlation with the abundance of all 6 types of immune cells in STAD (all *p* < 0.001). (**B**) NLRP3 expression is significantly negatively related to tumor purity and has a significant positive correlation with 5 types of immune cells (CD8+ T cells, CD4+ T cells, macrophages, neutrophils, and dendritic cells, other than B cells) in STAD (*p* < 0.001). (**C**) In six types of immune cell, the infiltration level of macrophage was significantly correlated with the cumulative survival of patients of STAD (*p* = 0.004).

The tumor microenvironment contains various infiltrating immune cells, which may affect patient prognosis [34]. Analysis of immune infiltrates in TIMER showed that NLRP1/NLRP3 expression levels were significantly negatively correlated with tumor purity and positively correlated with multiple infiltrating immune cells in STAD. Additionally, NLRP1 expression levels were significantly positively correlated with infiltrating levels of all six types of immune cells (all *p* < 0.001; Figure 5A). NLRP3 expression levels were significantly positively correlated with infiltrating levels of all immune cell types (all *p* < 0.001), except B cells, which were negatively correlated (r = −0.16, *p* = 0.002; Figure 5B). NLRP3 showed stronger correlations with CD8+ T cell, macrophages, neutrophils, and dendritic cells (all r > 0.4, *p* < 0.001) than did NLRP1. NLRP1 expression was positively correlated with B cell abundance, whereas NLRP3 expression was negatively correlated with B cell abundance. Additionally, STAD patients with high infiltration level of macrophages had a significant reduced survival (*p* = 0.004) (Figure 5C).

These results suggested that NLRP1/NLRP3 was associated with immune cell infiltration and high expression levels may promote tumor immune cell infiltration and leads to a poor prognosis. Moreover, NLRP1 and NLRP3 may play similar but not identical roles in immune-regulation in STAD.

### Relationships of NLRP1/NLRP3 with immune cell markers

**Figure 6:**
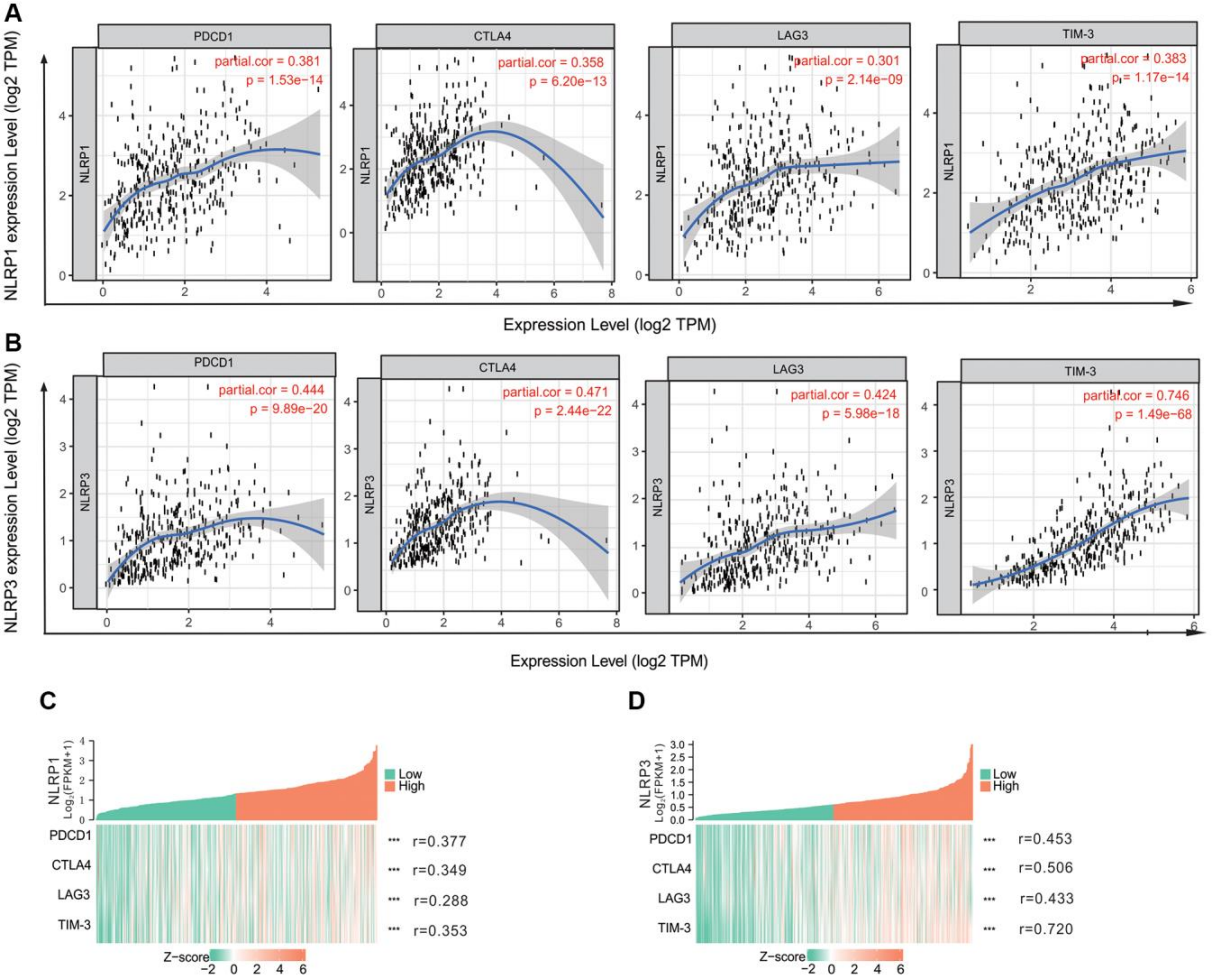
**The correlation of NLRP1/NLRP3 with immune checkpoints in TIMER 2.0.** (**A**) The expression scatterplots between NLRP1 and immune checkpoint genes (PCD1, r = 0.381, *p* = 1.53 × 10^−14^; CTLA4, r = 0.358, *p* = 6.20 × 10^−13^; LAG3, r = 0.301, *p* = 2.14 × 10^−9^; and TIM-3 r = 0.383, *p* = 1.17 × 10^−14^); (**B**) The expression scatterplots between NLRP3 and immune checkpoint genes (PCD1, r = 0.444, *p* = 9.89 × 10^−20^; CTLA4, r = 0.471, *p* = 2.44 × 10^−22^; LAG3, r = 0.424, *p* = 5.98 × 10^−18^; and TIM-3 r = 0.746, *p* = 1.49 × 10^−68^). (**C**) The single-gene co-expression heat map of NLRP1 and immune checkpoint genes (PCD1, r = 0.377, *p* < 0.001); CTLA4, r = 0.349, *p* < 0.001; LAG3, r = 0.288, *p* < 0.001; and TIM-3 r = 0.353, *p* < 0.001); (**D**) The single-gene co-expression heat map of NLRP3 and immune checkpoint genes (PCD1, r = 0.453, *p* < 0.001); CTLA4, r = 0.506, *p* < 0.001; LAG3, r = 0.433, *p* < 0.001; and TIM-3, r = 0.720, *p* < 0.001).

**Figure 7:**
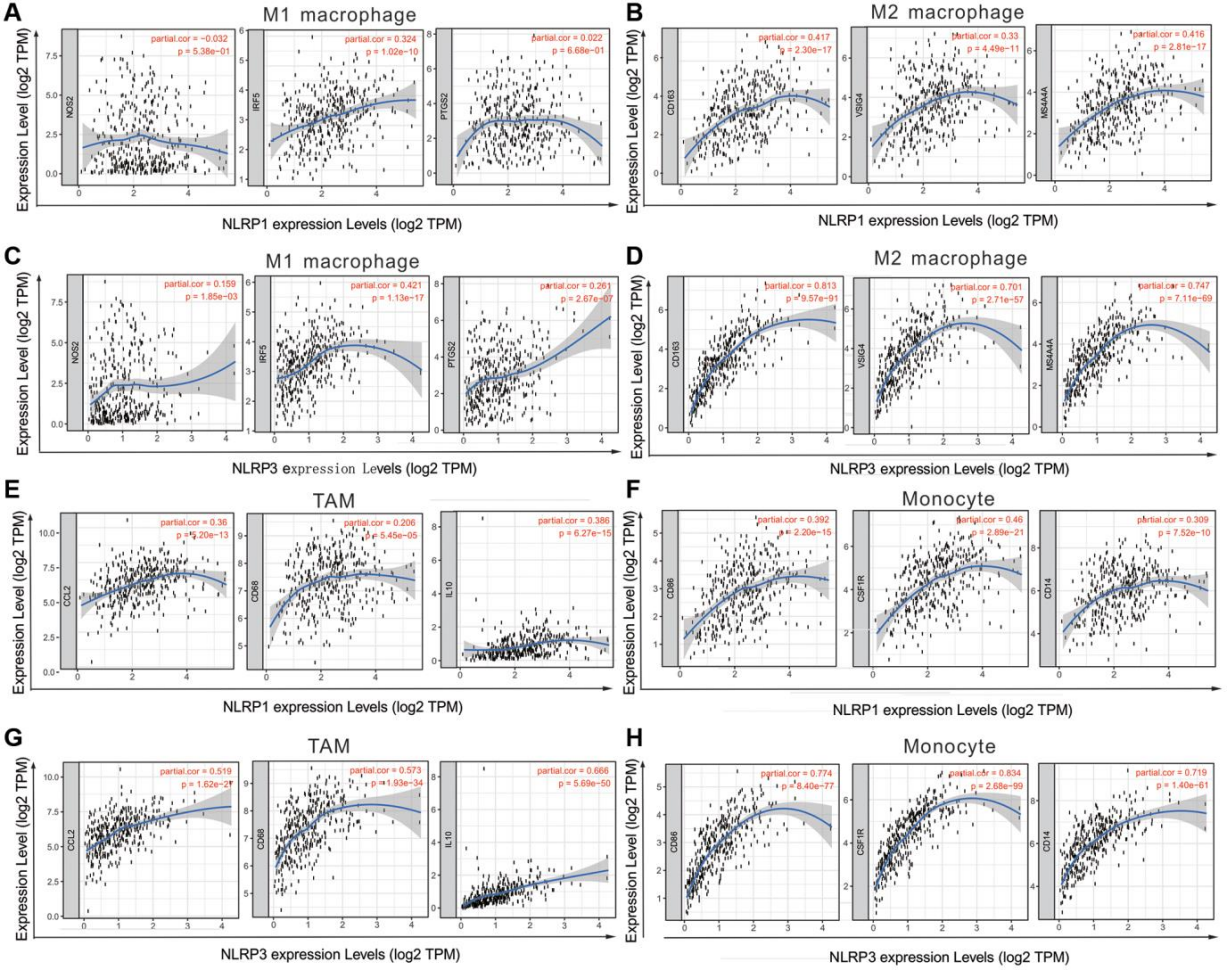
**NLRP1/NLRP3 expression level with macrophage polarization in STAD in TIMER 2.0 (*n* = 415).** The expression scatter-plots between NLRP1 and marker genes of (**A**) M1 macrophage, (**B**) M2 macrophage. The expression scatterplots between NLRP3 and marker genes of (**C**) M1 macrophage, (**D**) M2 macrophage. The expression scatter-plots between NLRP1 and marker genes of (**E**) TAM, and (**F**) monocyte. The expression scatterplots between NLRP3 and marker genes of (**G**) TAM, and (**H**) monocyte.

We assessed correlations of NLRP1/NLRP3 expression with 57 immune cell markers in STAD to explore the potential mechanisms through which NLRP1/NLRP3 modulate immune cell infiltration. NLRP1/NLRP3 expression levels were positively correlated with the vast majority of marker sets of various immune cells in STAD (Tables 2 and 3). NLRP1 was significantly positively correlated with the marker sets of T cells, B cells, regulatory T cells (Tregs), and M2 macrophages. As compared with NLRP1, NLRP3 had stronger positive correlations with a broader variety of immune cell markers. Specifically, for innate immune cells, NLRP3 expression was significantly positively correlated with almost all markers of monocytes, tumor-associated macrophages (TAMs), M2 macrophages, neutrophils, and dendritic cells (all r > 0.5, *p* < 0.0001). For specific immune cells, NLRP3 was significantly positively correlated with most markers of T cells, Tfh cells, Th1 cells, Th2 cells, Th17 cells, Tregs, and T-cell exhaustion, particularly with markers of T cell exhaustion (programmed cell death-1 [PD-1], cytotoxic T-lymphocyte antigen [CTLA] 4, lymphocyte activating 3 [LAG3], T cell immunoglobulin and mucin domain-containing protein 3 [TIM-3]; all r > 0.5, *p* < 0.0001) (Tables 3, Figure 6A and 6B), which are important immune checkpoints. The single-gene co-expression heat map showed similar results that the expression level of NLRP1/NLRP3 was significantly positively correlated with that of PD-1 (r = 0.453), CTLA4 (r = 0.506), LAG3 (r = 0.433), and TIM-3 (r = 0.720) (all *p* < 0.0001; Figure 6C and 6D). In addition, NLRP1 and NLRP3 expression had consistently weaker correlations with M1 macrophage marker sets, but consistently stronger correlations with marker sets of M2 macrophage, TAM, and Monocyte (Table 3, Figure 7A–7H), revealing that NLRP1/NLRP3 may induce TAM polarization towards M2 phenotype. All above results strongly suggested that NLRP1/NLRP3 may be involved in regulation of infiltration of immune cells in GC.

**Table 2:** Correlation analysis between NLRP1/NLRP3 and related genes and markers of innate immune cells in TIMER.

**Description**	**Genemarkers**	**NLRP1**				**NLRP3**			
		**None**		**Purity**		**None**		**Purity**	
		**Cor**	***P***	**Cor**	***P***	**Cor**	***P***	**Cor**	***P***
Monocyte	CD86	0.421	3.22e−19	0.392	2.20e−15	0.777	6.59e−85	0.774	8.40e−77
	CD115(CSF1R)	0.471	0e+00	0.460	2.89e−21	0.826	6.49e−105	0.834	2.68e−99
	CD14	0.342	8.02e−13	0.309	7.52e−10	0.729	5.38E−70	0.719	1.40e−61
TAM	CCL2	0.387	6.55e−17	0.360	5.20e−13	0.549	5.41e−34	0.519	1.62e−27
	CD68	0.217	8.88e−06	0.206	5.45e−05	0.581	7.39e−39	0.573	1.93e−34
	IL10	0.415	9.96e−19	0.386	6.27e−15	0.693	1.22e−60	0.666	5.69e−50
M1 macrophage	INOS(NOS2)	−0.02	6.92e−01	−0.032	5.38e−01	0.177	2.95e−04	0.159	1.85e−03
	IRF5	0.339	1.87e−12	0.324	1.02e−10	0.416	8.27e−19	0.421	1.13e−17
	COX2(PTGS2)	0.061	2.16e−01	0.022	6.68e−01	0.286	2.79e−09	0.261	2.67e−07
M2 macrophage	CD163	0.431	0e+00	0.417	2.30e−17	0.812	8.35e−99	0.813	9.57e−91
	VSIG4	0.34	1.42e−12	0.330	4.49e−11	0.697	1.16e−61	0.701	2.71e−57
	MS4A4A	0.434	0e+00	0.416	2.81e−17	0.749	1.07e−75	0.747	7.11e−69
Neutrophils	CD66b (CEACAM8)	0.078	1.12e−01	0.072	1.62e−01	0.127	9.46e−03	0.140	6.42e−03
	CD11b (ITGAM)	0.524	0e+00	0.515	4.83e−27	0.736	5.46e−72	0.729	6.02e−64
	CCR7	0.586	0e+00	0.567	1.33e−33	0.627	8.42e−47	0.608	1.03e−39
Natural killer cell	KIR2DL1	0.257	1.14e−07	0.229	6.70e−06	0.334	2.79e−12	0.339	1.27e−11
	KIR2DL3	0.174	3.75e−04	0.145	4.76e−03	0.287	2.61e−09	0.278	3.61e−08
	KIR2DL4	0.158	1.21e−03	0.109	3.40e−02	0.257	1.09e−07	0.215	2.48e−05
	KIR3DL1	0.293	1.24e−09	0.274	6.06e−08	0.305	2.27e−10	0.298	3.13e−09
	KIR3DL2	0.252	2.01e−07	0.222	1.31e−05	0.325	1.24e−11	0.311	6.02e−10
	KIR3DL3	−0.034	4.85e−01	−0.043	4.09e−01	0.117	1.75e−02	0.137	7.56e−03
	KIR2DS4	0.195	6.53e−05	0.156	2.40e−03	0.240	7.25e−07	0.250	8.50e−07
Dendritic cell	HLA-DPB1	0.418	0e+00	0.384	9.95e−15	0.578	2.07e−38	0.556	4.03e−32
	HLA-DQB1	0.298	7.59e−10	0.244	1.48e−06	0.418	5.77e−19	0.379	2.30e−14
	HLA-DRA	0.362	3.82e−14	0.327	6.88e−11	0.573	1.47e−37	0.559	1.78e−32
	HLA-DPA1	0.352	2.11e−13	0.315	3.47e−10	0.569	5.12e−37	0.553	9.49e−32
	BDCA-1(CD1C)	0.522	1.94e−30	0.514	6.48e−27	0.540	1.02e−32	0.504	8.43e−26
	BDCA-4(NRP1)	0.515	0e+00	0.499	2.88e−25	0.700	2.22e−62	0.690	9.06e−55
	CD11c (ITGAX)	0.487	0e+00	0.471	2.46e−22	0.810	1.22e−97	0.802	1.61e−86

Abbreviations: Cor: R value of Spearman’s correlation; None: correlation without adjustment. Purity, correlation adjusted by purity.

**Table 3:** Correlation analysis between NLRP1/NLRP3 and related genes and markers of adaptive immune cells in TIMER.

**Description**	**Gene markers**	**NLRP1**				**NLRP3**			
		**None**		**Purity**		**None**		**Purity**	
		**Cor**	***P***	**Cor**	***P***	**Cor**	***P***	**Cor**	***P***
B cell	CD19	0.527	4.63e−31	0.512	1.04e−26	0.385	4.44e−16	0.35	2.43e−12
	CD79A	0.473	0e+00	0.441	1.97e−19	0.413	1.57e−18	0.376	3.84e−14
T cell	CD3D	0.444	0e+00	0.409	1.06e−16	0.512	3.61e−29	0.481	2.18e−23
	CD3E	0.472	0e+00	0.436	4.82e−19	0.524	1.27e−30	0.496	6.24e−25
	CD2	0.477	0e+00	0.448	4.58e−20	0.597	2.15e−41	0.583	6.40e−36
CD8-T cell	CD8A	0.449	0e+00	0.418	1.77e−17	0.513	2.74e−29	0.495	7.68e−25
	CD8B	0.317	4.62e−11	0.304	1.55e−09	0.343	7.26e−13	0.324	9.74e−11
Tfh	IL21	0.268	3.06e−08	0.256	4.17e−07	0.393	8.31e−17	0.368	1.44e−13
	BCL6	0.457	9.07e−23	0.436	5.54e−19	0.439	5.57e−21	0.420	1.36e−17
Th1	T-bet (TBX21)	0.514	2.45e−29	0.491	2.17e−24	0.558	2.34e−35	0.538	7.04e−30
	STAT4	0.586	0e+00	0.569	7.54e−34	0.664	4.67e−54	0.660	9.38e−49
	STAT1	0.261	7.77e−08	0.258	3.46e−07	0.355	9.19e−14	0.363	2.93e−13
	IFN-γ (IFNG)	0.218	7.23e−06	0.192	1.73e−04	0.336	2.04e−12	0.325	8.50e−11
	TNF-α (TNF)	0.232	1.94e−06	0.178	5.39e−04	0.399	2.8e−17	0.351	1.87e−12
Th2	GATA3	0.433	0e+00	0.409	1.12e−16	0.414	1.27e−18	0.402	3.73e−16
	STAT6	0.266	3.76e−08	0.308	9.11e−10	0.280	6.18e−09	0.300	2.64e−09
	STAT5A	0.497	2.97e−27	0.479	3.51e−23	0.571	3.3e−37	0.571	3.23e−34
	IL13	0.178	2.7e−04	0.190	1.93e−04	0.157	1.37e−03	0.162	1.58e−03
Th17	STAT3	0.393	0e+00	0.390	3.52e−15	0.549	5.2e−34	0.554	6.68e−32
	IL17A	−0.085	8.42e−02	−0.096	6.12e−02	0.031	5.35e−01	0.007	8.84e−01
Treg	FOXP3	0.426	0e+00	0.393	1.96e−15	0.575	8.1e−38	0.558	1.88e−32
	CCR8	0.474	1.18e−24	0.464	1.23e−21	0.683	2.58e−58	0.683	1.94e−53
	STAT5B	0.519	0e+00	0.535	1.68e−29	0.552	1.87e−34	0.568	8.17e−34
	TGF (TGFB1)	0.461	3.56e−23	0.437	4.51e−19	0.516	1.49e−29	0.505	5.70e−26
T cell exhaustion	PD-1 (PDCD1)	0.411	0e+00	0.381	1.53e−14	0.459	5.13e−23	0.444	9.89e−20
	CTLA4	0.392	0e+00	0.358	6.20e−13	0.497	3.03e−27	0.471	2.44e−22
	LAG3	0.333	4.36e−12	0.301	2.14e−09	0.440	4.36e−21	0.424	5.98e−18
	TIM-3 (HAVCR2)	0.407	0e+00	0.383	1.17e−14	0.747	3.33e−75	0.746	1.49e−68
	GZMB	0.207	2.27e−05	0.158	2.06e−03	0.359	4.3e−14	0.328	5.78e−11

### GSEA

**Figure 8:**
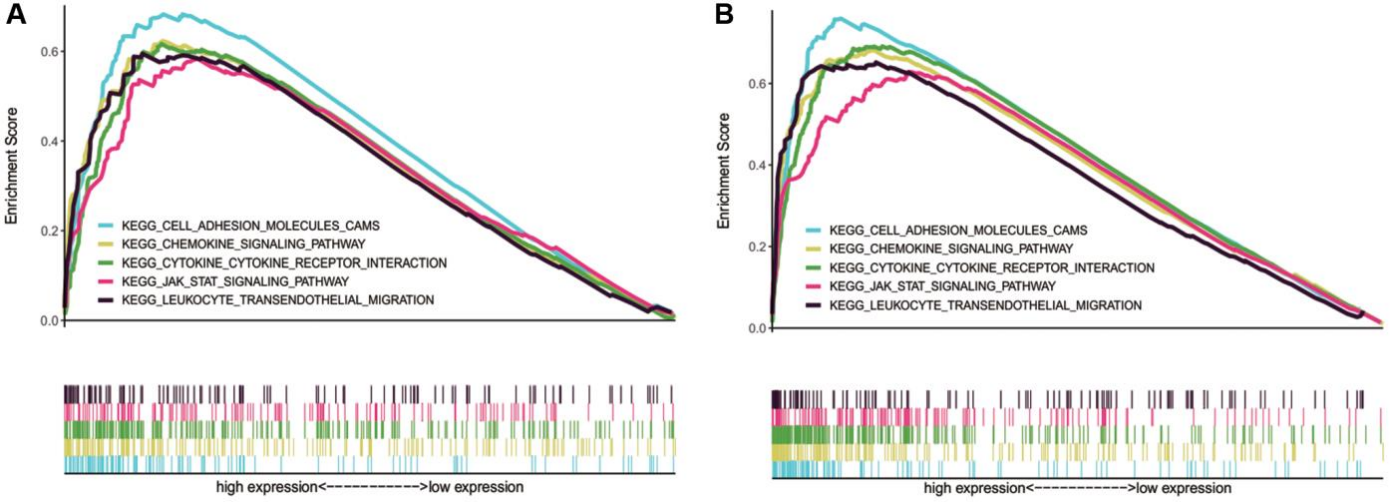
**Tumor immune-related pathways enriched in NLRP1/NLRP3 high NLRP1/NLRP3 expression group by using GSEA.** (**A** and **B**) NLRP1 and NLRP3 were both involved in pathways of cell adhesion molecules (CAMs), chemokines, leukocyte transendothelial migration, cytokine-cytokine receptor interaction, and leukocyte transendothelial migration.

GSEA was conducted to increase our understanding of the biological pathways in which NLRP1/NLRP3 may be involved. The results showed that NLRP1 and NLRP3 were both involved in some tumor immune-related signaling pathways, including pathways of chemokines, leukocyte transendothelial migration, cell adhesion molecules (CAMs), cytokine-cytokine receptor interaction, mitogen-activated protein kinase (MAPK), Janus kinase (JAK)/signal transducer and activator of transcription (STAT) (Table 4, Figure 8A and 8B), suggesting NLRP1/NLRP3 may participate in the same tumor immune-related pathways to regulate tumor immune cell infiltration. Details of other related pathways can be found in the Supplementary Tables 2, 3 and Supplementary Figures 1–10.

**Table 4:** Gene sets of tumor immune-related enriched in NLRP1/NLRP3 high expression Group.

**NLRP1**					**NLRP3**				
**NAME**	**SIZE**	**NES**	**NOM *p*-val**	**FDR *q*-val**	**NAME**	**SIZE**	**NES**	**NOM *p*-val**	**FDR *q*-val**
KEGG_CHEMOKINE_SIGNALING_PATHWAY	125	2.282	0.000	0.009	KEGG_CHEMOKINE_SIGNALING_PATHWAY	183	2.414	0.000	0.000
KEGG_LEUKOCYTE_TRANSENDOTHELIAL_MIGRATION	183	2.255	0.000	0.004	KEGG_LEUKOCYTE_TRANSENDOTHELIAL_MIGRATION	115	2.341	0.000	0.007
KEGG_CYTOKINE_CYTOKINE_RECEPTOR_INTERACTION	115	2.204	0.000	0.005	KEGG_CYTOKINE_CYTOKINE_RECEPTOR_INTERACTION	254	2.308	0.000	0.006
KEGG_JAK_STAT_SIGNALING_PATHWAY	150	2.156	0.002	0.006	KEGG_JAK_STAT_SIGNALING_PATHWAY	150	2.299	0.000	0.005
KEGG_CELL_ADHESION_MOLECULES_CAMS	125	2.089	0.000	0.007	KEGG_CELL_ADHESION_MOLECULES_CAMS	125	2.239	0.000	0.004
KEGG_TOLL_LIKE_RECEPTOR_SIGNALING_PATHWAY	254	2.084	0.000	0.007	KEGG_FC_GAMMA_R_MEDIATED_PHAGOCYTOSIS	91	2.225	0.000	0.004
KEGG_MAPK_SIGNALING_PATHWAY	264	2.059	0.000	0.008	KEGG_TOLL_LIKE_RECEPTOR_SIGNALING_PATHWAY	100	2.192	0.000	0.004
KEGG_T_CELL_RECEPTOR_SIGNALING_PATHWAY	107	2.041	0.000	0.007	KEGG_T_CELL_RECEPTOR_SIGNALING_PATHWAY	107	2.153	0.000	0.003
KEGG_B_CELL_RECEPTOR_SIGNALING_PATHWAY	75	2.034	0.000	0.007	KEGG_PATHWAYS_IN_CANCER	321	2.098	0.000	0.004
KEGG_FC_GAMMA_R_MEDIATED_PHAGOCYTOSIS	91	2.019	0.000	0.008	KEGG_MAPK_SIGNALING_PATHWAY	264	2.094	0.000	0.004

Gene sets with NOM *p*-value < 0.01, FDR *q*-value < 0.01 and NES > 2.0 are considered. Note: SIZE: total number of genes under this gene set. Abbreviations: NES: normalized enrichment score; NOM: nominal; FDR: false discovery rate.

## DISCUSSION

Early inflammation is a defense mechanism against cancer cells, and inflammasomes are closely related to tumor progression, prognosis, and treatment response [17–19]. In this study, we reported the potential value of NLRP1/NLRP3 as a prognostic marker of GC and provided preliminary evidence of the relationship between NLRP1/NLRP3 and immune cell infiltration into GC and the potential underlying mechanisms. These results provided bioinformatics-based evidence for the application of NLRP1/NLRP3 in predicting prognosis and selecting the appropriate immunotherapy in patients with GC and improved our understanding of the roles of NLRP1/NLRP3 in the GC regulatory network.

In our study, we first analyzed the differential expression of NLRP1/NLRP3 in various tumors and normal tissues based on chip data from the Oncomine database. We found that NLRP1 had opposite expression differences in two GC datasets, whereas NLRP3 showed no differential expression between tumor and normal tissues. Therefore, Oncomine analysis results were inconclusive in terms of determining the correlation between NLRP1/NLRP3 and GC. Consequently, we analyzed NLRP1/NLRP3 expression in different tumor types based on TCGA RNA-seq data. After comprehensive analysis, we found that both NLRP1 and NLRP3 were upregulated in GC as compared with normal control tissue, which was consistent with result of TIMER analysis. The difference of *P* values in the analysis results of the two databases may be related to the sample size of the database or the different data analysis methods. Subsequently, immunohistochemical and western blotting experiments using 20 tissue samples from patients with GC also confirmed the elevated expression of NLRP1/NLRP3 in GC. These results suggested that NLRP1 and NLRP3 were related to tumorigenesis and that high expression of NLRP1/NLRP3 may promote the occurrence and development of GC. Subsequently, Kaplan-Meier plotter microarray data combined with TCGA RNA-Seq data were used for survival analysis to evaluate the impact of NLRP1/NLRP3 expression levels on prognosis in patients with GC. Notably, our results showed that patients with GC with high expression of NLRP1/NLRP3 (particularly NLRP3) had significantly reduced survival. Furthermore, correlation analysis of NLRP1/NLRP3 with clinical features showed that high expression of NLRP1/NLRP3 correlated significantly with worse prognosis in GC patients with lymph node metastasis. In particular, NLRP3 showed a stronger correlation with poor prognosis in both databases. The high expression of NLRP3 in gastric cancer promotes the activation of NLRP3 inflammasome and the secretion of interleukin-1β (IL-1β) in macrophages. In addition, NLRP3 binds to the cyclin-D1 (CCND1) promoter and promotes its transcription in gastric epithelial cells. miR-22 is expressed in gastric mucosa and directly targets NLRP3 as an inhibitor of NLRP3 [34].

Recent reports have demonstrated that cytotoxin-associated gene A and mycoplasma hyorhinis promote the migration and invasion of GC cells by activating NLRP3, which is consistent with our findings [25, 26]. The above results indicated that high expression of NLRP1/NLRP3 may promote the invasion and metastasis of GC and lead to poor prognosis in patients with GC. Thus, NLRP1/NLRP3, particularly NLRP3, may be effective markers of GC prognosis. Although a few reports have described the correlations between NLRP1 and GC and our findings indicated its prognostic potential through bioinformatics analyses, experimental results are still needed to confirm these findings.

Tumor tissue is not comprised purely of tumor cells. Almost all types of immune cells have been found in the tumor microenvironment, and these cells secrete various factors that influence the tumor microenvironment and regulate tumor behavior [35]. Therefore, we analyzed the correlations between NLRP1/NLRP3 and infiltrating immune cells in STAD via the TIMER database. The scatter diagram showed that NLRP1/NLRP3 expression levels were positively correlated with the abundances of various infiltrating immune cells, suggesting that increased expression of NLRP1/NLRP3 may promote immune cell infiltration in STAD.

We further investigated the potential mechanisms through which NLRP1/NLRP3 were involved in tumor immune-regulation by analyzing the correlations between NLRP1/NLRP3 and immune cell marker genes. The results revealed NLRP1/NLRP3 (particularly NLRP3) were likely involved in inducing M2 macrophage polarization. We also found high infiltration levels of macrophages were significantly associated with reduced survival in patients with GC. Therefore, we speculated that NLRP1/NLRP3 may promote the progression of GC by regulating polarization of macrophages, thus leading to poor prognosis. Recently, NLRP3 has been reported to participate in the regulation of macrophage polarization through different pathways in some diseases, such as colitis [36], gouty arthritis [37], and hepatocellular carcinoma [38], whereas only a few studies have described the regulation of tumor-infiltrating macrophage polarization in GC. The mechanisms of NLRP3 regulation remain unclear.

In addition, positive correlations of NLRP1/NLRP3 with Treg and T-cell exhaustion markers indicated that NLRP1/NLRP3 may induce T-cell exhaustion by activating Tregs. Furthermore, NLRP1 and NLRP3 were consistently positively correlated with almost all T-helper cell markers, and the results were stronger for NLRP3 than for NLRP1. These correlations suggested that NLRP1/NLRP3, particularly NLRP3, may be involved in regulating T-cell functions. A recent study reported that NLRP3 promotes the differentiation of T helper 1 (Th1) cells [39], consistent with our results.

Immune checkpoints involve immunosuppressive molecules. High expression of these molecules can deplete T cells, thereby reducing immune surveillance, suppressing the killing of tumor cells, and eventually leading to immune escape of tumor cells [5]. In tumor immunotherapy, immune checkpoint inhibitors reactivate the antitumor immune response through co-inhibitory T-cell signal transduction, thus achieving antitumor effects. This therapy has shown unprecedented clinical efficacy in a variety of tumors [4, 7]. We also found that NLRP1/NLRP3 had positive correlations with some important immune checkpoint genes, such as *TIM-3*, *PD-1*, *CTLA4*, and *LAG3*. NLRP3 showed stronger correlations with immune checkpoint genes than did NLRP1 (Tables 3, Figure 6). For example, NLRP3 had the strongest correlation with TIM-3 (r > 0.7, *p* < 0.001). Several recent reports have shown that TIM-3 plays an important role in regulating dendritic cell function and inhibiting anti-tumor immunity by modulating NLRP3 inflammasome activation [40]. The NLRP3 inflammasome upregulates PD-L1 expression and participates in immunosuppression of lymphoma [41]. These results confirmed that NLRP1/NLRP3, particularly NLRP3, may modulate immune checkpoints and promote immune escape of tumor cells.

Because the mechanisms through which NLRP1 and NLRP3 regulate tumor immune infiltration in GC are not clear, we performed GSEA, which revealed the potential biological pathways involving NLRP1/NLRP3. The possible key immunity-related pathways included chemokine, leukocyte transendothelial migration, and CAMs. Chemokine expression is related to high tumor immunogenicity, T-cell infiltration, and the antitumor response [42–44]. The transendothelial migration of leukocytes is an essential step in leukocyte recruitment at inflammatory sites. Chemokines mediate cell adhesion and migration and play an important role in leukocyte migration. Leukocyte migration and changes in the surrounding microenvironment precisely regulate a cascade of events, including the development of diseases, such as cancer [45, 46]. CAMs are involved in the binding of cells to the extracellular matrix and regulate the immune response of cells [47]. In addition, NLRP1/NLRP3 may also be involved in several important tumor-related pathways, such as MAPK and JAK-STAT pathways [48, 49]. Therefore, NLRP1/NLRP3 may regulate immune infiltration in GC through these immune- and tumor-related pathways. However, because of a lack of studies of the mechanisms through which NLRP1 and NLRP3 regulate tumor immune cell infiltration in GC, more experimental studies are needed.

## CONCLUSIONS

In conclusion, our study comprehensively analyzed the correlations of the NLRP1/NLRP3 inflammasome with GC prognosis and immune cell infiltration using multiple databases. Elevated expression of NLRP1/NLRP3 was found to be related to poor prognosis in patients with GC. Furthermore, NLRP1/NLRP3 may be involved in macrophage polarization, T-cell exhaustion, Tregs, immune checkpoint regulation, and other tumor immune regulatory processes. Therefore, NLRP1/NLRP3, particularly NLRP3, may play key roles in immune regulation and may serve as biomarkers for prognosis in patients with GC. Because the roles of NLRP1/NLRP3 in immune invasion in GC are affected by various cytokines in the tumor microenvironment, further *in vitro* and *in vivo* studies are needed to elucidate the related mechanisms. The prognostic value of NLRP1/NLRP3 in GC also needs to be confirmed by further experimental data with larger sample sizes.

## Supplementary Materials

Supplementary Figures

Supplementary Table 1

Supplementary Table 2

Supplementary Table 3
