# Supplementary material for: The prognostic value of NLRP1/NLRP3 and its relationship with immune infiltration in human gastric cancer
Source: Aging (Albany NY). 2022 Dec 19;14(24):9980–10008. doi: 10.18632/aging.204438 (PMC9831740; doi:10.18632/aging.204438)
Supplement: Supplementary Figures [file aging-14-204438-s001.pdf]

SUPPLEMENTARY FIGURES

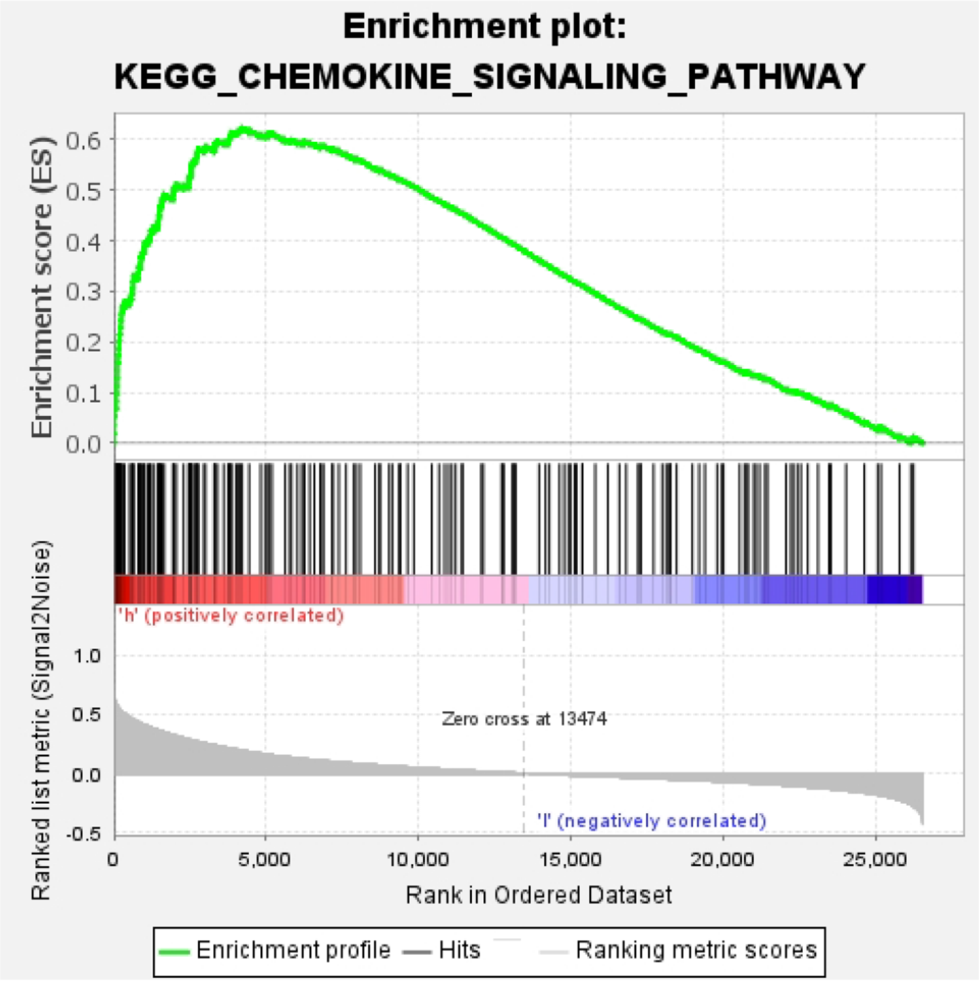

Supplementary Figure 1. NLRP1 was involved in pathways of chemokines.

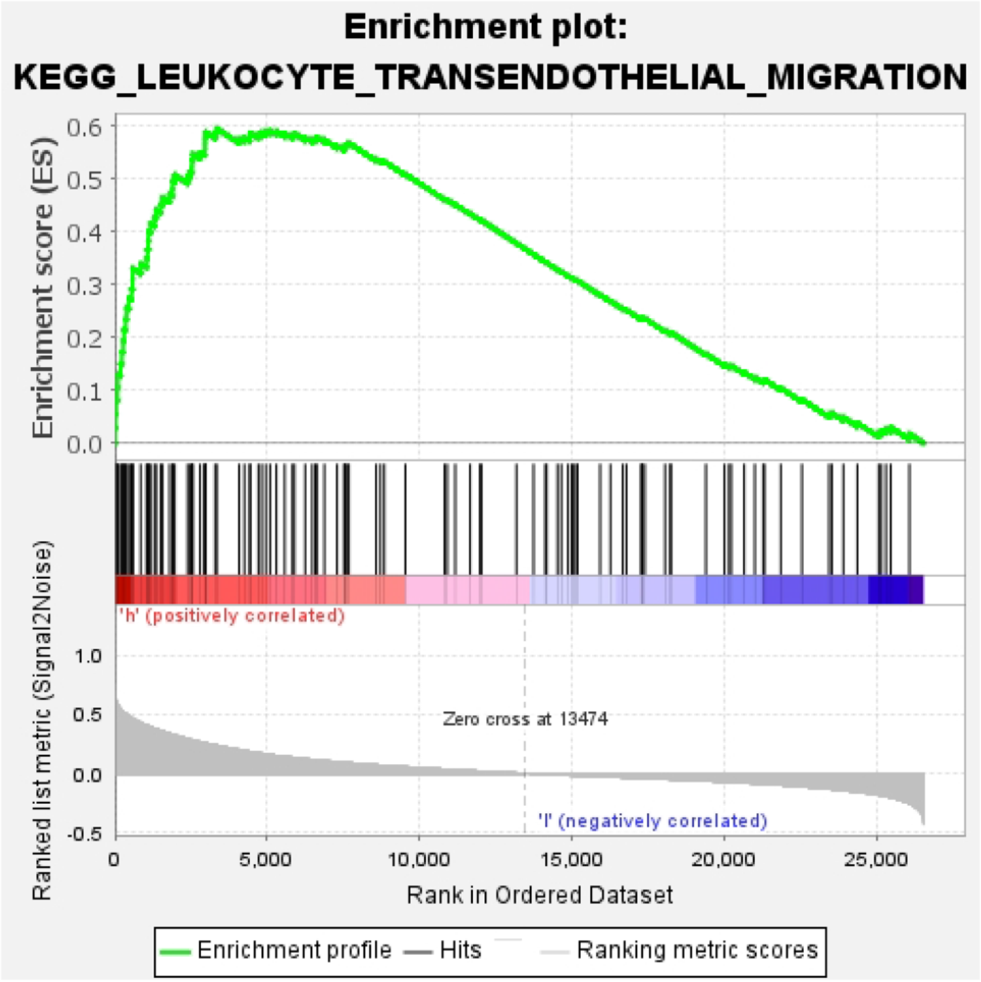

Supplementary Figure 2. NLRP1 was involved in pathways of leukocyte transendothe-lial migration.

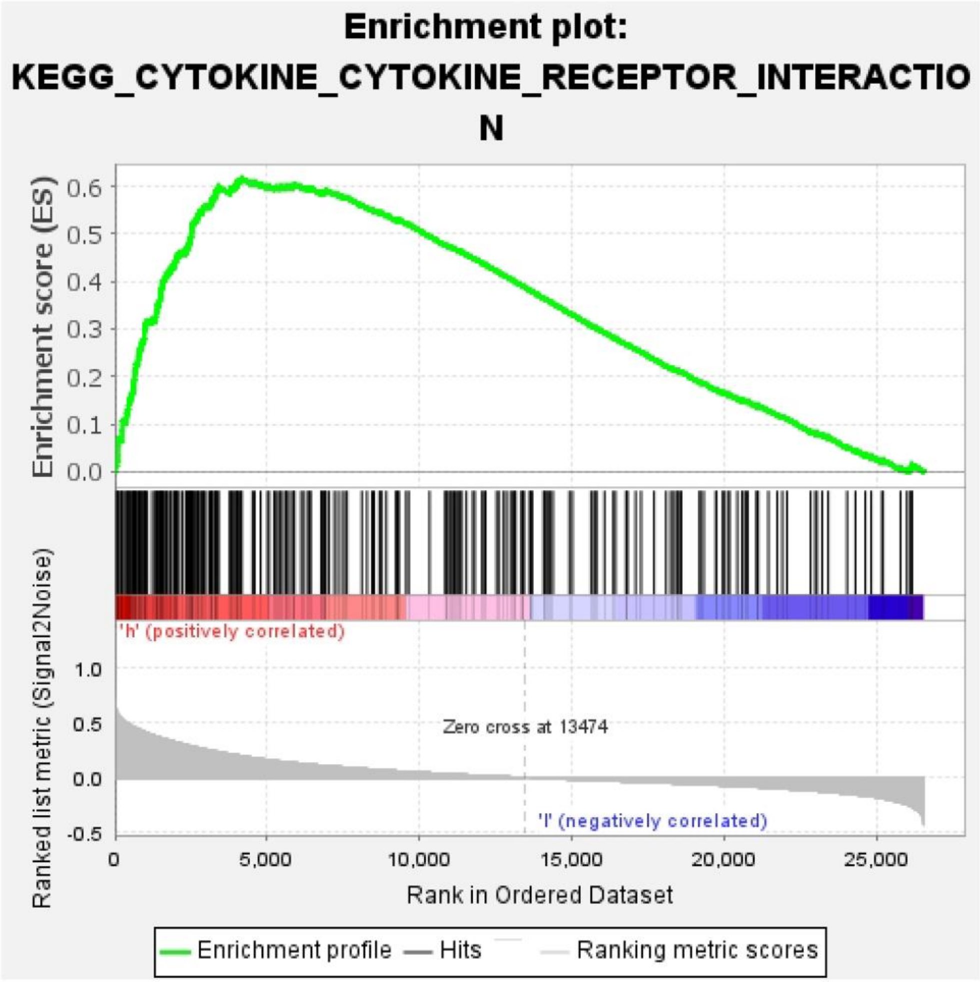

Supplementary Figure 3. NLRP1 was involved in pathways of cytokine-cytokine receptor interaction.

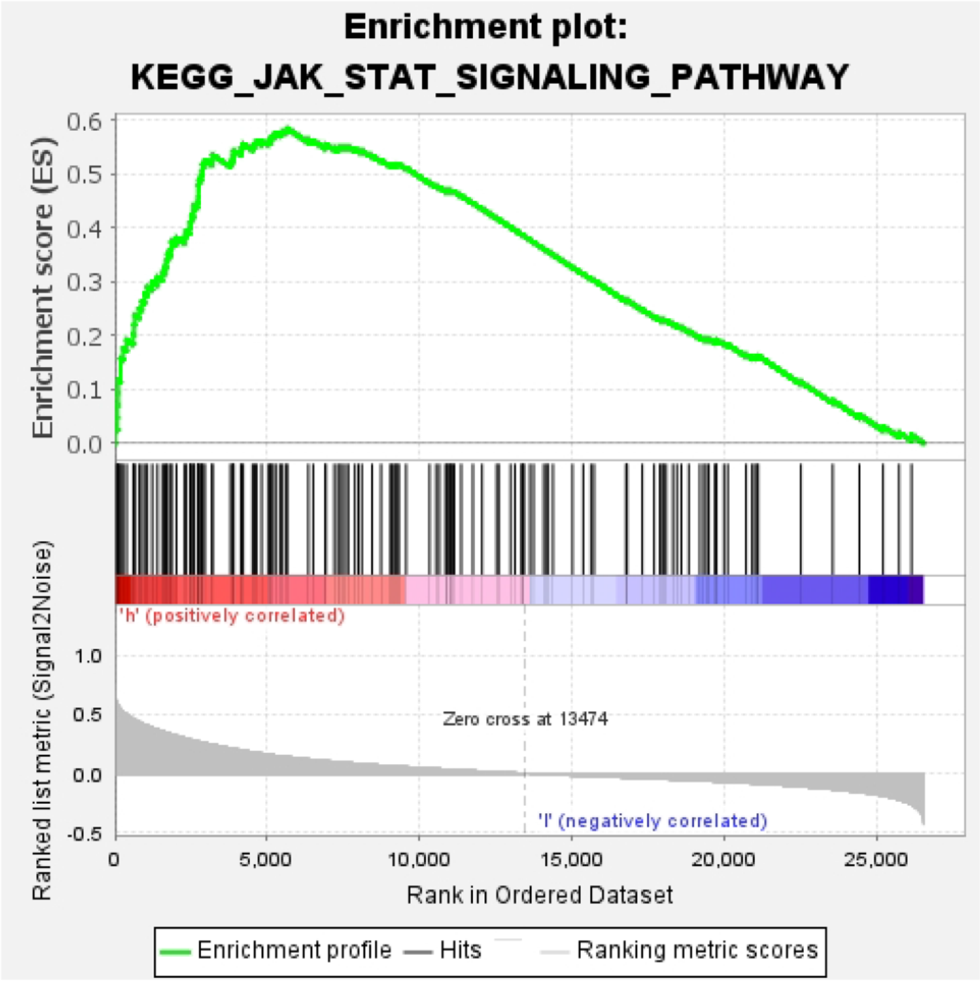

Supplementary Figure 4. NLRP1 was involved in pathways of leukocyte transendothelial migration.

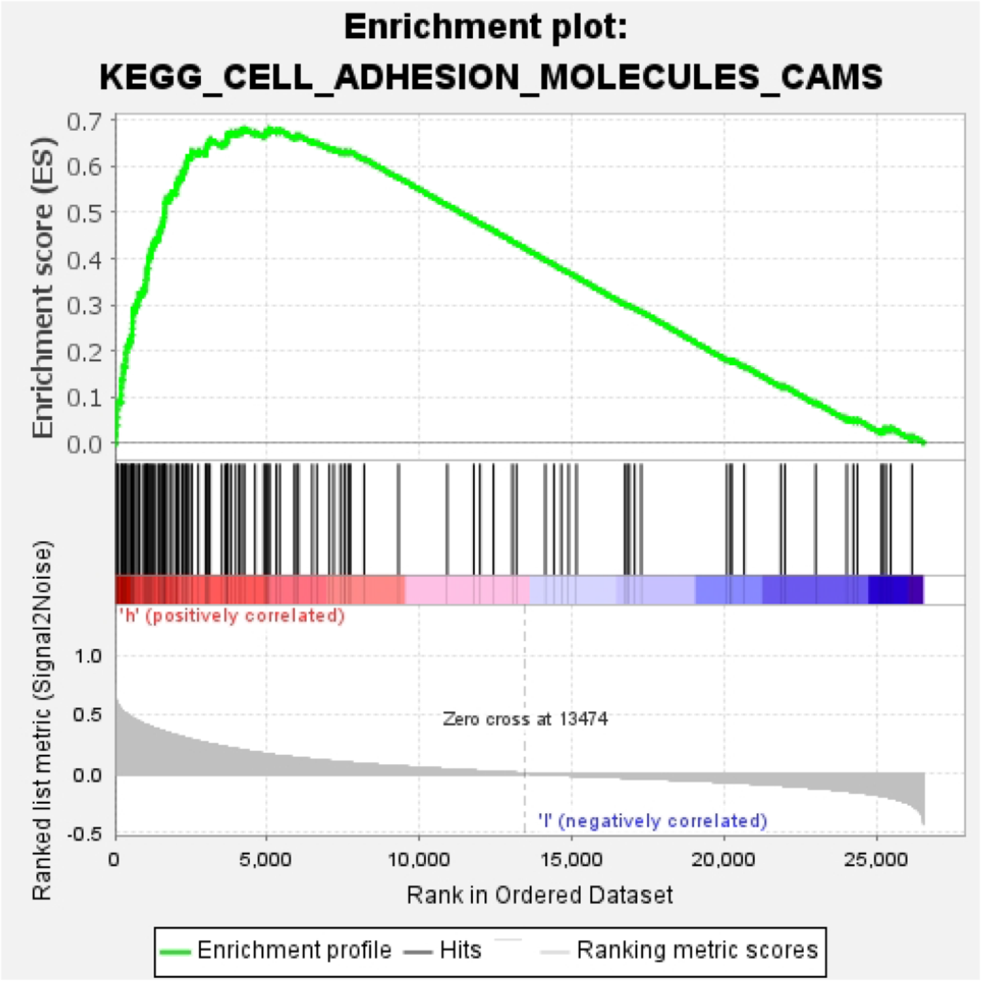

Supplementary Figure 5. NLRP1 was involved in pathways of cell adhesion molecules (CAMs).

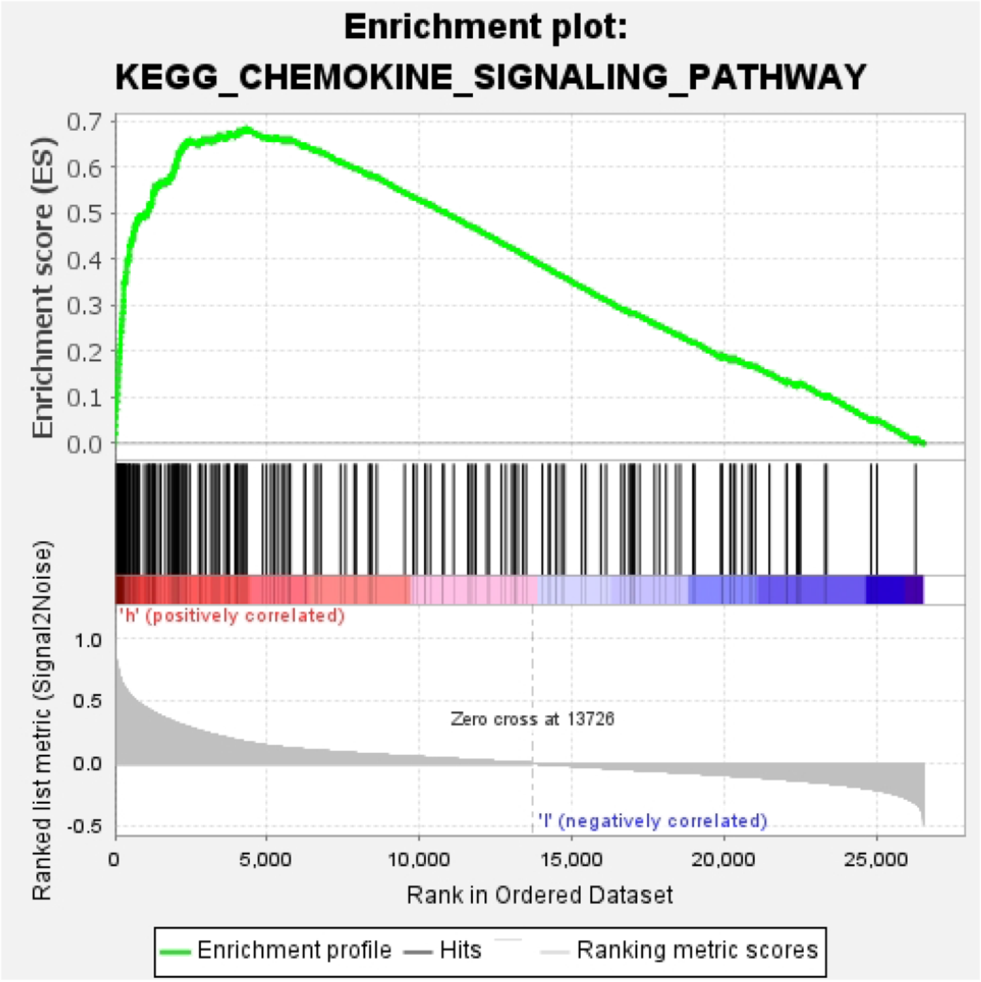

Supplementary Figure 6. NLRP3 was involved in pathways of chemokines.

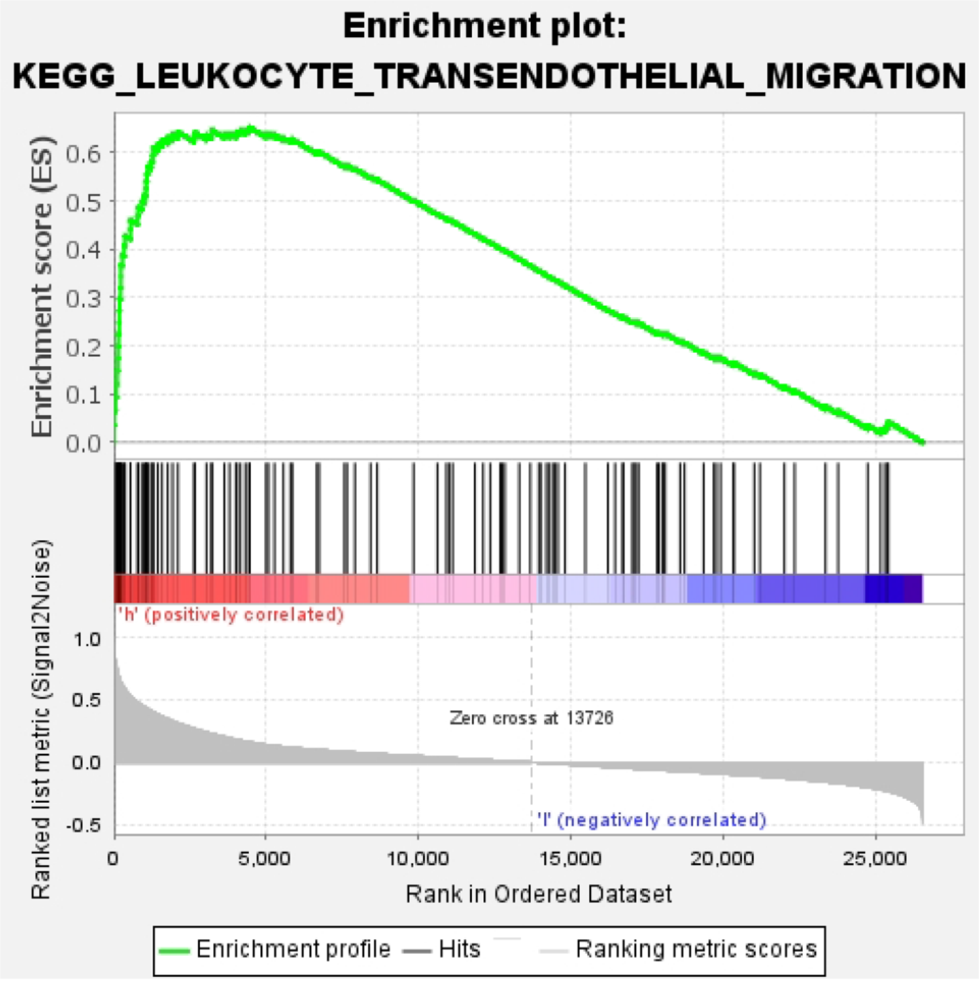

Supplementary Figure 7. NLRP3 was involved in pathways of leukocyte transendothe-lial migration.

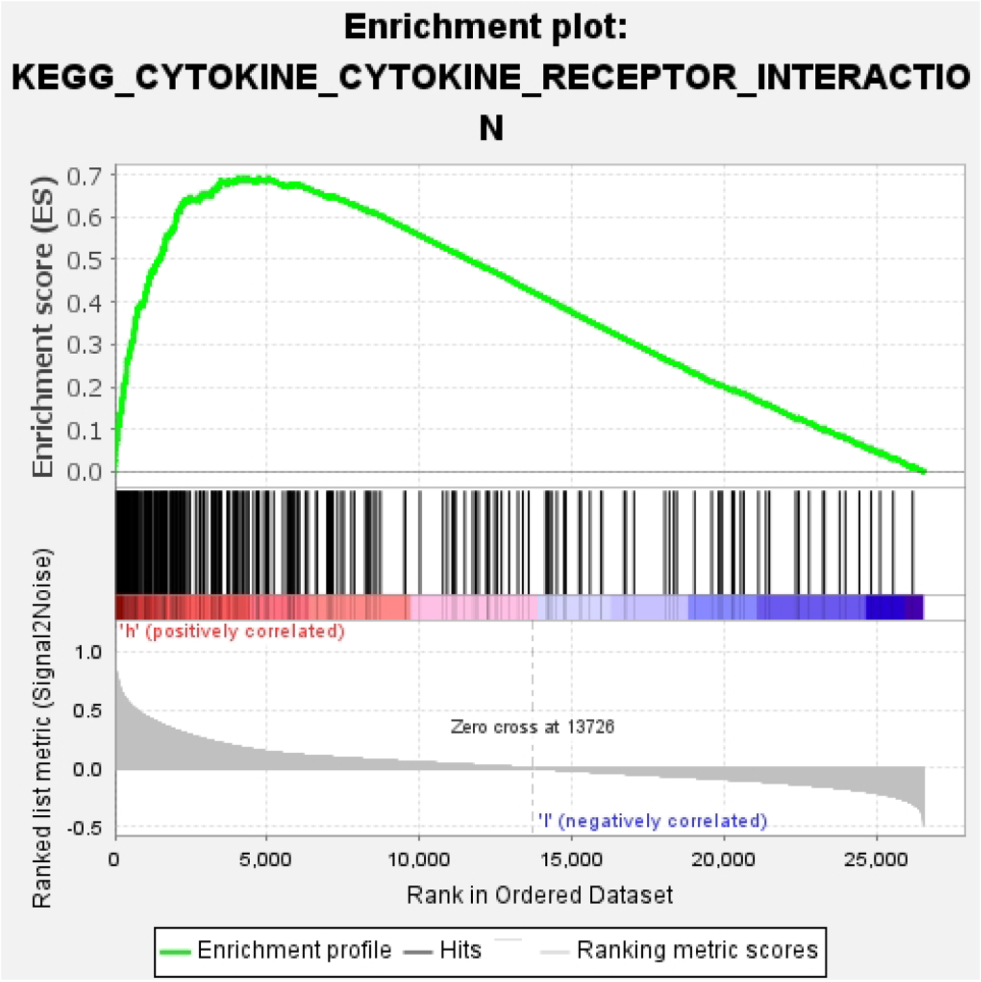

Supplementary Figure 8. NLRP3 was involved in pathways of cytokine-cytokine receptor interaction.

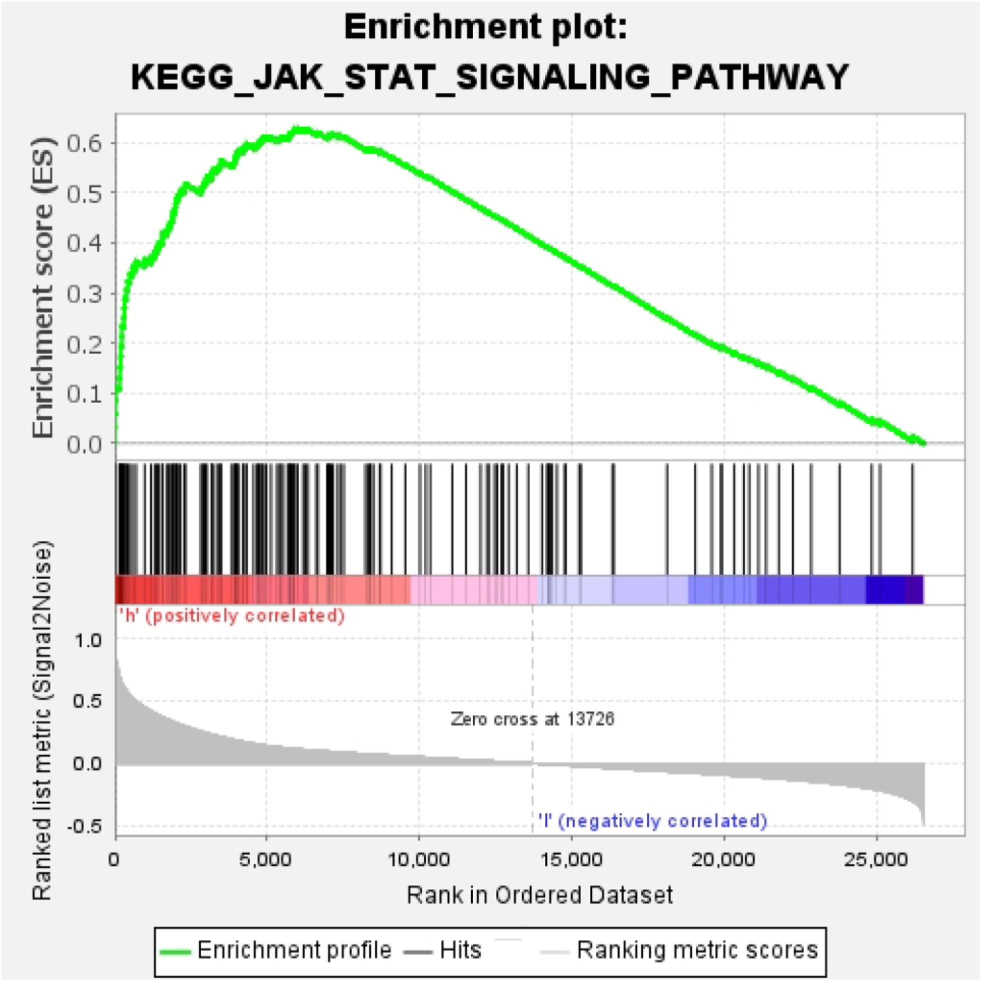

Supplementary Figure 9. NLRP3 was involved in pathways of leukocyte transendothelial migration.

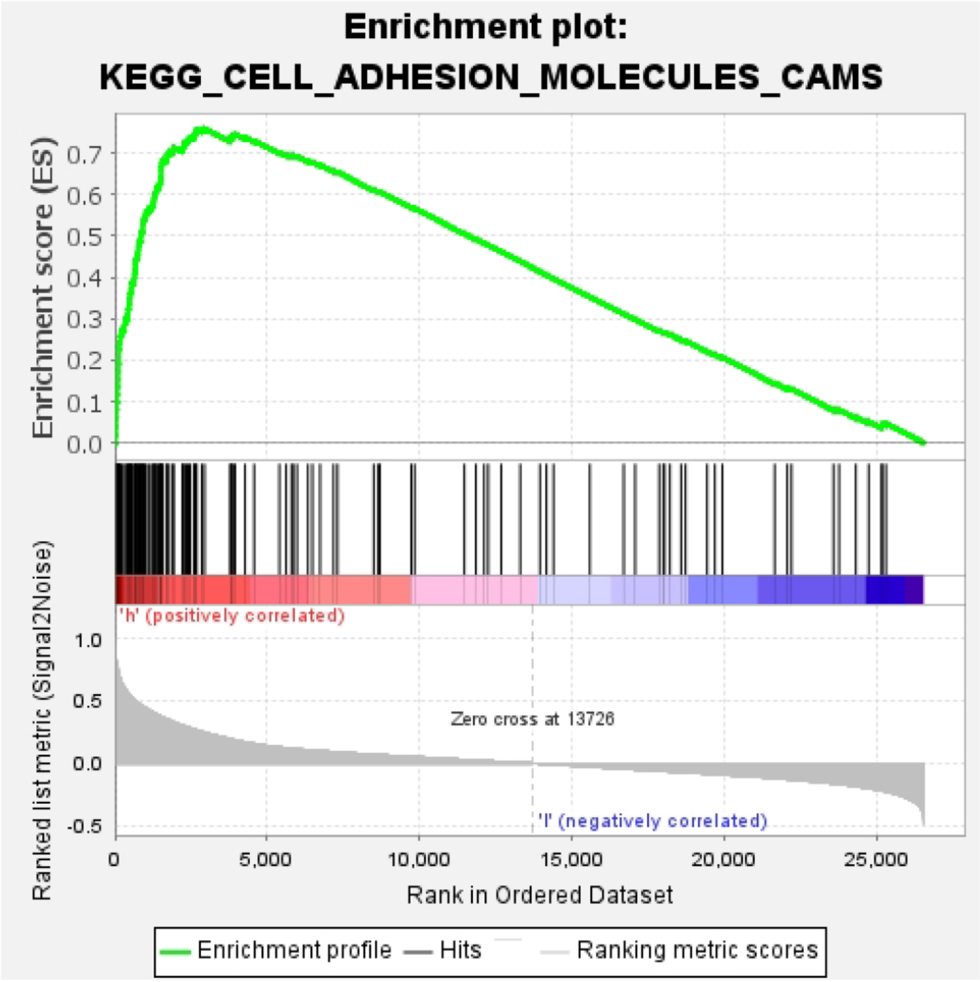

Supplementary Figure 10. NLRP3 was involved in pathways of cell adhesion molecules (CAMs).
