# Supplementary material for: The prognostic value of NLRP1/NLRP3 and its relationship with immune infiltration in human gastric cancer
Source: Aging (Albany NY). 2022 Dec 19;14(24):9980–10008. doi: 10.18632/aging.204438 (PMC9831740; doi:10.18632/aging.204438)
Supplement: Supplementary Table 1 [file aging-14-204438-s002.pdf]

SUPPLEMENTARY TABLE

Supplementary Table 1. Clinical information of 20 patients with gastric cancer.

No.	Age	Gender	Clinical diagnosis	Tumors size status	Histological grading degree of differentiation	Lymph node stasus	TNM staging
1	56	male	Gastric antrum cancer	5.5 × 4	Poorly differentiated gastric adenocarcinoma	9/23	T1N2M0
2	85	male	Gastric cancer	6 × 5	Poorly differentiated gastric adenocarcinoma	24/25	T3N3M0
3	72	male	Gastric cardia cancer	2 × 3	Poorly differentiated gastric adenocarcinoma	14/18	T3N3M1
4	72	male	Gastric cardia cancer	5 × 6	Gastric neuroendocrine carcinoma	1/14	T3N1M0
5	63	male	Gastric antrum cancer	5 × 4	Gastric neuroendocrine carcinoma	1/22	T3N1M0
6	70	male	Gastric antrum cancer	3 × 3	Moderately-*differentiated adenocarcinoma*	7/20	T3N3M0
7	76	male	Gastric antrum cancer	7 × 4	Poorly differentiated gastric adenocarcinoma	5/28	T4N2M0
8	74	male	Gastric cardia cancer	12 × 7	Poorly differentiated gastric adenocarcinoma	6/22	T3N2M0
9	63	male	Gastric cancer	4 × 3	Poorly differentiated gastric adenocarcinoma	12/30	T3N3M0
10	73	female	Gastric cancer	7 × 5	Moderately-*differentiated adenocarcinoma*	34/44	T3N3M0
11	78	male	Gastric cardia cancer	5 × 4	Mucinous adenocarcinomas	10/22	T3N3M0
12	65	male	Gastric antrum cancer	3 × 3	Moderately-*differentiated adenocarcinoma*	1/25	T3N1M0
13	64	male	Gastric antrum cancer	4 × 4	Moderately-*differentiated adenocarcinoma*	4/19	T1N2M0
14	77	male	Gastric cardia cancer	3 × 3	Moderately-*differentiated adenocarcinoma*	1/26	T3N1M0
15	75	female	Gastric cancer	10 × 8	Signet-ring *cell* carcinoma.	5/10	T3N2M0
16	69	female	Gastric cancer	5 × 4	Moderately-*differentiated adenocarcinoma*	4/16	T2N2M0
17	65	male	Gastric antrum cancer	3 × 3	moderately-*differentiated adenocarcinoma*	9/9	T3N3M0
18	72	male	Gastric antrum cancer	4 × 3	moderately-*differentiated adenocarcinoma*	3/26	T1N2M0
20	62	male	Gastric cancer	3 × 3	poorly differentiated gastric adenocarcinoma	3/33	T3N2M0
21	71	female	Gastric cancer	3 × 3	moderately-*differentiated adenocarcinoma*	11/15	T2N3M0
22	77	male	Gastric cardia cancer	4 × 4	Signet-ring *cell* carcinoma.	5/24	T3N3M0
23	73	female	Gastric cancer	8 × 6	Moderately-*differentiated adenocarcinoma*	16/28	T3N3M0
